# Perturbation of Quorum Sensing after the Acquisition of Bacteriophage Resistance Could Contribute to Novel Traits in *Vibrio alginolyticus*

**DOI:** 10.3390/microorganisms11092273

**Published:** 2023-09-10

**Authors:** Dimitrios Skliros, Stavros Droubogiannis, Chrysanthi Kalloniati, Pantelis Katharios, Emmanouil Flemetakis

**Affiliations:** 1 Laboratory of Molecular Biology, Department of Biotechnology, School of Applied Biology and Biotechnology, Agricultural University of Athens, 11855 Athens, Greece; dskliros@aua.gr (D.S.); xkalloni@gmail.com (C.K.); 2 Institute of Marine Biology, Biotechnology and Aquaculture, Hellenic Centre for Marine Research, 71500 Heraklion, Greece; stavros.drou@gmail.com (S.D.); katharios@hcmr.gr (P.K.); 3 Department of Marine Sciences, University of the Aegean, 81100 Mytilene, Greece

**Keywords:** bacteriophages, quorum sensing, phage resistance, biofilm, virulence, vibrio

## Abstract

Bacteria employ a wide range of molecular mechanisms to confer resistance to bacteriophages, and these mechanisms are continuously being discovered and characterized. However, there are instances where certain bacterial species, despite lacking these known mechanisms, can still develop bacteriophage resistance through intricate metabolic adaptation strategies, potentially involving mutations in transcriptional regulators or phage receptors. *Vibrio* species have been particularly useful for studying the orchestrated metabolic responses of Gram-negative marine bacteria in various challenges. In a previous study, we demonstrated that *Vibrio alginolyticus* downregulates the expression of specific receptors and transporters in its membrane, which may enable the bacterium to evade infection by lytic bacteriophages. In our current study, our objective was to explore how the development of bacteriophage resistance in *Vibrio* species disrupts the quorum-sensing cascade, subsequently affecting bacterial physiology and metabolic capacity. Using a real-time quantitative PCR (rt-QPCR) platform, we examined the expression pattern of quorum-sensing genes, auto-inducer biosynthesis genes, and cell density regulatory proteins in phage-resistant strains. Our results revealed that bacteriophage-resistant bacteria downregulate the expression of quorum-sensing regulatory proteins, such as *LuxM*, *LuxN*, and *LuxP*. This downregulation attenuates the normal perception of quorum-sensing peptides and subsequently diminishes the expression of cell density regulatory proteins, including *LuxU*, *aphA*, and *LuxR*. These findings align with the diverse phenotypic traits observed in the phage-resistant strains, such as altered biofilm formation, reduced planktonic growth, and reduced virulence. Moreover, the transcriptional depletion of *aphA*, the master regulator associated with low cell density, was linked to the downregulation of genes related to virulence. This phenomenon appears to be phage-specific, suggesting a finely tuned metabolic adaptation driven by phage–host interaction. These findings contribute to our understanding of the role of *Vibrio* species in microbial marine ecology and highlight the complex interplay between phage resistance, quorum sensing, and bacterial physiology.

## 1. Introduction

Interactions among microbes in the marine habitat are known to be multidimensional, complex, and challenging to study [1]. It is well established that these interactions shape genetic variability and metabolic capacity [2]. Marine prokaryotic viruses, such as bacteriophages, have emerged as key players in shaping microbial diversity, influencing the phenotypic traits of their natural enemies [3]. Bacteriophages contribute significantly to the global geochemical cycle of carbon and nitrogen, causing the lysis of over 20% of microbes daily [4]. In response, bacteria develop complex molecular and biochemical countermeasures, which affect their normal physiology and can lead to strain diversification, particularly in marine species [5]. This metabolic shift towards bacteriophage tolerance often results in diverse fitness traits, which are only now being explored and understood. When considering the use of bacteriophages as antimicrobial agents, it is essential to investigate acquired phage resistance or tolerance for potential negative phenotypes, such as altered growth, biofilm formation, or even virulence [6]. Bacteriophage resistance can involve well-documented molecular mechanisms within the cell [7] or a metabolic adaptation strategy triggered by genomic mutations, commonly observed in Gram-negative bacteria [8]. Our previous study demonstrated that aquatic species, such as vibrios, can enhance their tolerance towards lytic bacteriophages, by downregulating the expression pattern of membrane transporters [9]. This study revealed the cellular and metabolic cost of the acquired phage resistance at genomic, transcriptomic, and metabolomic levels, subsequently impacting various phenotypic traits in *V. alginolyticus* in a phage-specific manner. These findings raised new questions about the fate and role of quorum sensing (QS) following the acquisition of phage resistance.

Vibrios are frequently involved in studies exploring the molecular mechanism of QS [10]. The association between autoinducer perception, uptake, synthesis, and bacterial lifestyle is well documented [11]. Many traits have been attributed to QS in vibrios, including growth rate, biofilm formation, and virulence. It is now evident that bacteria regulate behavioral traits in a complex manner, associated with autoinducer abundance under continuously changing environmental and microenvironmental niches [12]. Understanding how the presence and interaction with lytic bacteriophage influences the intricate quorum-sensing (QS) system is an area that requires further exploration. The impact of bacteriophages on QS-related processes in vibrios can shed light on the interplay between these key regulatory mechanisms and the dynamics of microbial communities.

*Vibrio alginolyticus* is a rod-shaped marine bacterium. In the past, numerous bacteriophages have been isolated as potential therapeutic agents from this species as it is an important fish pathogen [13,14,15]. *V. alginolyticus* can biosynthesize at least three quorum-sensing (QS) peptides: acylated homoserine lactones (AHLs) or AI-1, furanosyl borate diester or AI-2, and cholerae autoinducer-1 or CAI-1. These peptides play a crucial role in coordinating the bacterium’s metabolic processes, virulence, and biofilm formation [16]. The two key regulators that define trait decisions between virulence and biofilm or of the population during a changing environment in vibrios are *aphA* and *LuxR* [17,18]. *aphA* is considered as the master regulator for genes involved during low-cell density (LCD) state, while *LuxR* is the master regulator of genes involved during high-cell density (HCD) state [17]. In the presented study, we used bacteriophage-resistant mutants of two lytic bacteriophages for exploring the fate of the QS metabolic pathway and its presumable relationship with newly acquired phenotypic traits in the abundant marine species *V. alginolyticus*, particularly during exponential cell growth conditions, which are reflected as a low-cell density (LCD) state.

## 2. Materials and Methods

### 2.1. Vibrio Alginolyticus Strain V1

The strain of *V. alginolyticus* (V1) used in the study had been isolated previously from diseased gilthead seabream (*Sparus aurata*) in Crete, Greece, and has been fully sequenced [19]. The strain was routinely grown in Luria–Bertani (LB) broth (Tryptone 10 g/L; NaCl 10 g/L; Yeast extract 5 g/L) supplemented with 1 mM MgSO_4_ and 1 mM CaCl_2_ at 25 °C.

### 2.2. Bacteriophages φSt2 and Athena1

Two lytic bacteriophages were used in the current study (Table 1), both classified as members of the *Caudoviricetes* class and characterized by a myovirus morphotype. These bacteriophages primarily target *V. alginolyticus* strain V1 and were previously isolated from coastal regions of Greece. Extensive analysis has been conducted on their biological and molecular characteristics. Their biological characteristics have been studied and reported under 25 °C, which is an optimal temperature for *V. alginolyticus* growth. Despite both having a myovirus morphotype and similar time of latent period and burst size, these bacteriophages differ in terms of genome size. Bacteriophage *φ*St2 possesses a genome size of 250,485 bps including several genes that have been previously suggested to be involved in the modulation of key bacterial metabolic processes related to phage proliferation, such as the NAD^+^ biosynthesis cascade and the ribonucleotide metabolism [20], whereas Athena1 has a smaller genome size of 39,826 bps [21] and do not harbor any of these metbolic pathways.

### 2.3. Generating Phage-Resistant Mutants

Bacteriophage-resistant mutants were obtained through an experimental procedure involving overnight exposure to Athena1 at a multiplicity of infection (MOI) of 100 as described in Skliros et al. [9]. Briefly, three separate cultures of *V. alginolyticus* were grown in LB medium and inoculated with each phage during the exponential phase (OD_600_: ~0.2) at a MOI of 100. The cultures were then incubated overnight at 25 °C. After the overnight incubation, dilutions of the resulting phage lysates were prepared and placed on LB medium supplemented with 1.5% agar, 1 mL/L of 1 M MgSO_4_ and 1 mL/L of 1 M CaCl_2_. Following another overnight incubation at 25 °C, individual colonies were isolated from each plate. All selected colonies were subsequently cultured in LB broth and subjected to phage infection by using the spot test assay. To confirm the development of resistance, high-titer phage stocks of Athena1 (>10^9^) were employed. The phage-resistant colonies were further propagated by re-plating, and one colony from each plate was selected as a phage-resistant biological replicate and preserved at −80 °C with 20% glycerol for subsequent analysis. For *φ*St2 bacteriophage, resistant strains developed earlier [9] were used.

### 2.4. Growth Curve, Biofilm Formation, and Virulence

To generate growth curves, three independent resistant isolates for each bacteriophage and three wild-type strains were cultured in LB broth at 25 °C. During the exponential phase, they were transferred to a 96-well plate (TrueLine) with a volume of 200 μL, using three technical replicates for each resistant strain. The optical density at 600 nm was measured every 10 min using a plate reader (TECAN Infinity series 200 PRO, Männedorf, Switzerland) at 25 °C with shaking. For the microtiter plate biofilm formation capacity assay, the method described by O’Toole and Kolter [23] was followed with some modifications. *V. alginolyticus* biofilm formation was initiated by adding 950 μL of bacterial cells (OD_600_ = 0.3) and 50 μL of LB broth to tissue culture polystyrene 24-well microtiter plates (Costar 3524, Corning, NY, USA) at 25 °C for 24 h. Three independent colonies of both wild-type and phage-resistant strains for each bacteriophage were used, with 10 technical replicates for each colony. Biofilm formation was monitored by measuring the optical density at 540 nm (OD_540_) after staining with Crystal Violet solution, following the method by O’Toole and Kolter [23], using a microplate reader (TECAN Infinity series 200 PRO, Männedorf, Switzerland).

Bacterial virulence was assessed using an in vivo challenge model with gilthead seabream larvae as described previously [24]. Gilthead Seabream eggs at a similar developmental stage obtained from broodstock and kept in Hellenic Center of Marine Research (HCMR) facilities were utilized. The eggs underwent three washes with sterile seawater and were subsequently placed individually in six 96-well microplate, with each well containing 180 µL of sterile seawater. Following one day of incubation, the egg quality was assessed based on Panini et al. [25]. The virulence assay commenced upon larval hatching. Bacterial suspensions (20 μL) adjusted to approximately 10^7^ CFU/mL were added at each well resulting in a final concentration of 10^6^ cfu/mL. The bacteria tested had been previously cultured in LB medium overnight, then diluted 1:100 in fresh LB medium. Following a 2 h incubation at 25 °C, they were centrifuged washed twice with sterile saline water (0.9% NaCl).

Six different treatments were employed: a negative control with 20 μL of saline (0.9% NaCl) substituted for bacteria, a positive control utilizing the wild-type strain of *V. alginolyticus* (V1), and two biological replicates for each resistant strain. Fish larval survival was monitored daily for 5 days, and a Kaplan–Meier survival curve was subsequently constructed using Graphpad Prism.

### 2.5. DNA Sequencing of Phage-Resistant Mutants

A sodium dodecyl sulfate (SDS)-based protocol with protease treatment was employed to extract bacterial DNA from the obtained resistant strains for each bacteriophage. The following steps were performed. Harvested cells during the exponential phase were washed and then incubated at 56 °C for 2 h in DNA extraction buffer (2% SDS, 200 μg/mL proteinase K, Tris 10 mM pH 7.2, EDTA 0.1 mM, and 0.1% beta-mercaptoethanol). Afterwards, 7 μL of 30 mg/mL RNAse A (ThermoFisher Scientific, Waltham, MA, USA) was added to each sample and incubated at 37 °C for 20 min. DNA purification was performed using DNA purification silica columns from a commercially available kit (Macherey-Nagel, Duren, Germany) following the manufacturer’s protocol. DNA quality and RNA contamination were assessed using a 0.7% agarose gel and a Nanodrop spectrophotometer (ThermoFisher Scientific, Waltham, MA, USA). For the construction of a paired-end library and subsequent sequencing, at least 5 μg of high-purity bacterial DNA was used for each resistant strain. The DNA quality was evaluated using a BioAnalyzer (BioRad, CA, USA). A Nextera Library Construction Kit (Illumina, San Diego, CA, USA) was utilized with an insert size of ~350 bp to generate a paired-end library. Paired-end 300 genomic library sequencing was performed on the DNA samples using Illumina sequencing technology, with a minimum genome sequence depth of 300X to have increased confidence on base calling and detect possible single nucleotide polymorphisms (SNPs). Variant motifs (single nucleotides, insertions, and deletions) were searched with a minimum variant frequency of 0.25, a maximum variant *p*-value of 10^−6^, and a minimum strand-bias *p*-value of 10^−5^.

### 2.6. Targeted Gene Expression Analysis of Bacteriophage-Resistant Strains

To study gene expression, wild-type (phage-sensitive) and phage-resistant bacterial colonies were retrieved from −80 °C stocks and grown overnight at 25 °C with vigorous shaking in LB broth. In the present study, the resistant strains that were generated by Skliros et al. [9] for *φ*St2 were also used. Bacterial cells were recultured until an LCD state (OD_600_: 0.2) was achieved, and at the same time, a small aliquot was retrieved to re-validate their phage-resistant status with a spot assay. RNA extraction was performed using Nucleozol^TM^ (Macherey-Nagel, Düren, Germany) reagent according to the manufacturer’s protocol. The extracted RNA was treated with TURBO DNAse (Ambion, Austin, TX, USA) to remove DNA contaminants, and PCR was performed to verify the absence of bacterial DNA. The remaining RNA was checked for integrity using 2% agarose gel electrophoresis. PrimeScript RT reagent synthesis kit (Takara, Dalian, China), which utilizes the reverse transcription efficiency of the Primescript^TM^ enzyme, was employed for cDNA synthesis. Specifically, reverse-strand cDNA synthesis was performed in a 10 μL total reaction volume containing 800 ng total RNA, 0.5 μL PrimeScript™ RT Enzyme mix, 2 μL random hexamers (100 μM), PrimeScript Buffer for real-time PCR, and the addition of RNase-free ddH_2_O up to 10 μL. A master mix of the reagents was prepared before the addition of RNA in each sample. The reverse transcription occurred by utilizing the following protocol: 37 °C for 15 min, 85 °C for 7 s, and 4 °C for 5 min. Primer pairs for cDNA amplification (Table S1) were designed using the sequenced genome of the *V. alginolyticus* strain V1 and by utilizing Geneious software (R10 version; Biomatters Ltd., Auckland, New Zealand) [26] and were in silico tested against bacterial and bacteriophage genomic DNA. Quantitative real-time PCR was performed on a StepOnePlus™ Real-Time PCR System (Applied Biosystems, Foster City, CA, USA) using SYBR Select Master Mix (Applied Biosystems, Austin, TX, USA) up to 10 μL per reaction. The gene-specific primers were used at a final concentration of 0.2 μΜ each. In total, 1 μL of cDNA template was used per reaction. PCR cycling started with initial polymerase activation at 95 °C for 10 min, followed by 40 cycles at 95 °C for 15 s and 60 °C for 1 min. The formation of primer dimers and each primer’s specificity were monitored by dissociation curve analysis. Melt curves and 2% agarose gel for each reaction were monitored to ensure single product amplification. Two reference genes were used for increasing confidence to the transcriptional data. The expression levels of *V. alginolyticus* gyrase A subunit (*gyrA*; forward primer: CGGTACTGAGCAGATCCCAG and reverse primer: ACCAGAAGCACCGTTAACCA; Average Reaction Efficiency: 1.998; r^2^: 0.999) and the HSP70 protein (*dnaK*; forward primer: TCCTACACGTGTCTGCGAAA and reverse primer: CCGCCAGAAGCTTGGATAGT; Average Reaction Efficiency: 1.999; r^2^: 0.999) were used as reference genes to normalize cDNA templates and to compute the relative transcript level for each gene of interest, which was calculated as E^−ΔCt^. All cDNAs were diluted accordingly to appear a similar (±1.5 cycle) geometrical mean of the two reference genes before rt-qPCRs of the targeted genes occur. In every rt-qPCR 96-well plate run, both of the reference genes were also included. Ct difference between the two reference genes remained relatively stable among all cDNAs used in the study. ΔCt was calculated as Ct_X_–Ct_R_, where Ct_X_ corresponds to the Ct of the gene of interest, and Ct_R_ is the geometrical mean of the two HK genes’ Cts. PCR efficiency(E) for each amplicon was calculated from the slope of each reaction by applying the linear regression method to the log (fluorescence) per cycle number data, using LinRegPCR software (version 7.5) [27]. An average E for each primer pair was used.

Reference genes used during the transcriptional study were chosen based on previous transcriptional works on *V. alginolyticus* during exponential growth [28], studies on QS-related genes [29], and transcriptional studies relative to *Vibrio* sp. virulence [30]. The primers of the reference genes have also been used previously in transcriptional studies of *V. alginolyticus* strain V1 [9,20].

### 2.7. Statistical Analysis and Figures

Statistical analysis was performed using SigmaPlot software (version 14.0; Systat Software Inc., San Jose, CA, USA), where we utilized Student’s *t*-test for analysis of Statistical significance. All results passed normality test. If equal variance test failed, a Welch’s test was conducted. The results were plotted, and figure legends provide details on statistical significance, number of biological replications, and significance threshold. Survival analysis for the in vivo challenge test was conducted in GraphPad using the Kaplan–Meier method. Schematic representation Figures were created with BioRender.com (22 June 2023).

## 3. Results

### 3.1. Phenotypic Traits of Phage-Resistant Mutants

**Figure 1:**
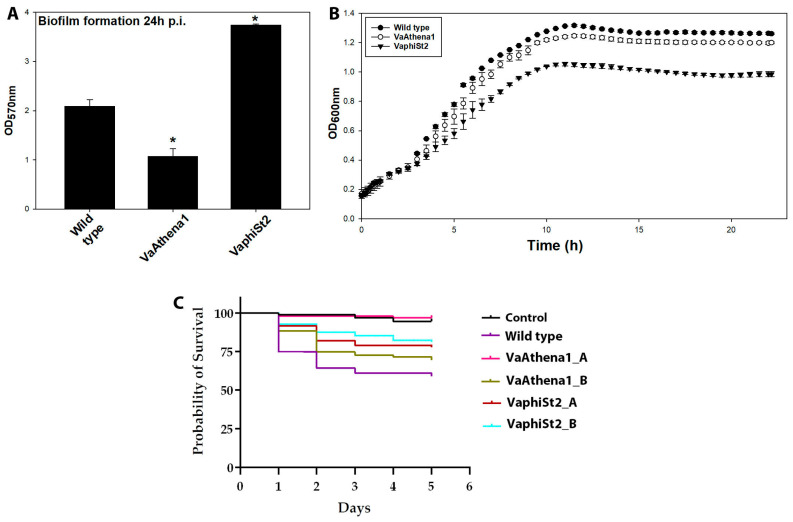
Phenotypic traits of wild-type susceptible strain, resistant strains to Athena1 (VaAthena1), and resistant strains to *φ*St2 (VaphiSt2). (**A**) For biofilm formation capacity after 24 h of post-inoculation (n = 3 independent colonies with 10 technical replicates; ±SE; *p* ≤ 0.05, post hoc Student’s *t*-test). (**B**) For growth rate (n = 3 for wild-type and n = 3 independent colonies with three technical replicates for phage-resistant strains; ±SE). (**C**) For a 5-day trial on fish larvae survivability of wild-type and two different resistant colonies of each lytic bacteriophage (n = 96). Asterisks show statistical significance compared to Wild type strain of *V. alginolyticus (p* ≤ 0.05).

After the generation of bacteriophage-resistant mutants, we proceeded in studying cross-resistance among them, which revealed that phage-resistant strains to *φ*St2 were also resistant to Athena1 bacteriophage (Table 2). However, the phage-resistant strains to Athena1 did not exhibit resistance to *φ*St2. Biofilm formation assays demonstrated a low deviation between the independent resistant colonies for each bacteriophage, with the average showing that the resistant strains to Athena1 (VaAthena1) exhibited up to 60% statistically significant reduction in biofilm formation 24 h post-inoculation (Figure 1A). In contrast, the phage-resistant strains VaphiSt2 showed a statistically significant increase of at least 50% in biofilm formation compared to the control group of phage-susceptible bacteria. Interestingly, when studying planktonic cells through growth kinetics, no difference was observed in the case of VaAthena1-resistant strains, but a 20% decrease in growth rate was observed for VaphiSt2-resistant strains (Figure 1B). In regard to the virulence assay, we assessed the average survival probability of fish larvae for two individual colonies of each resistant strain.

The survival rate of fish larvae challenged with the wild-type (WT) *V. alginolyticus* strain (V1) was found to be 58.94% during the 5-day trial, in comparison to the negative control group where 99% of larvae survived. Both VaphiSt2 strains tested exhibited significantly lower virulence (*p* < 0.05) compared to the WT strain, with survival rates of 77.89% and 81.25%, respectively. Similarly, one of the two resistant strains against phage Athena1 displayed statistically lower virulence, resulting in a survival rate of 96.87% (*p* < 0.05), while the second strain tested showed a survival rate of 69.47%, which, although higher, was not statistically different from the survival rate observed in the larvae challenged with the WT strain (Figure 1C). Taken together, all these results exhibit a phenotypic alteration of the bacteriophage-resistant strains compared to the WT bacteria.

### 3.2. Transcript Profiling of QS Cassette

**Figure 2:**
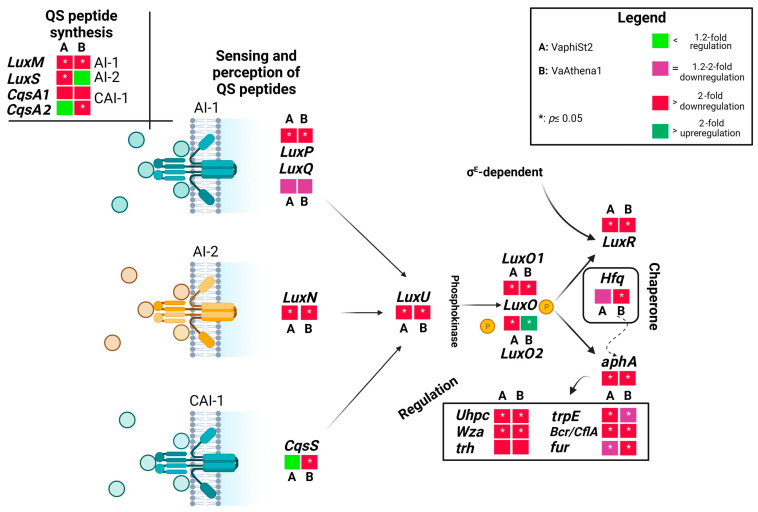
Schematic representation of the QS cascade, including QS perception, biosynthesis, and regulated genes under low cell density. Figure shows fold changes in the relative transcript abundances of genes of bacteriophage-resistant strains VaphiSt2 and VaAthena1, compared to wild-type strains demonstrated as heat maps. Black arrows represent the QS pathway and sigma factors responsible for the transcription of QS-related genes. The dotted arrow represents chaperone activity. In the case of genes *CqsA* and *LuxO*, two distinct isoforms were annotated and studied (n = 3; post hoc Student’s test applied for normalized values with *p* ≤ 0.05; Tukey test applied for non-normalized values).

**Figure 3:**
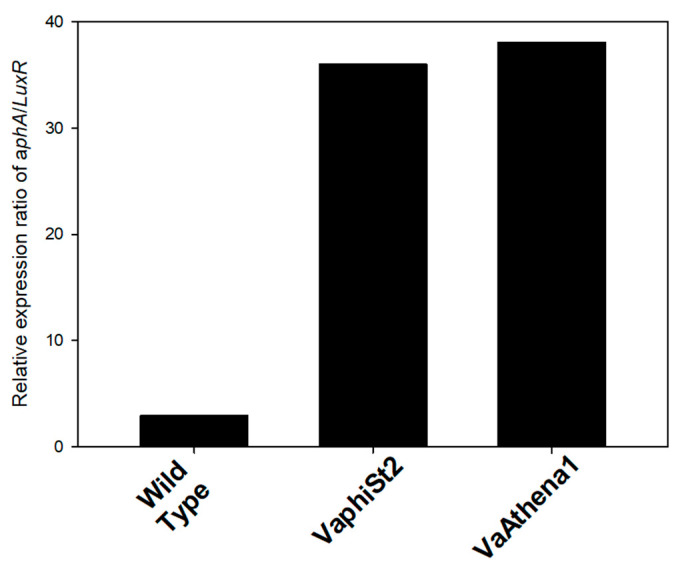
Relative expression ratio of *aphA/LuxR* of the resistant strains VaphiSt2 and VaAthena1, as well as wild-type susceptible strains.

In an attempt to gain insights into the metabolic reprograming that the cells are experiencing with an emphasis on the fate and the contribution of QS reception and perception among the *V. alginolyticus* populations of the wild-type and the resistant strains, we employed a targeted RT-qPCR analysis (Figure 2; Table S2). We utilized phage-resistant strains for Athena1 bacteriophage obtained here and phage-resistant strains to *φ*St2 generated in [9]. To this end, we determined the relative transcript levels for 14 genes involved in perception, regulation, and biosynthesis of QS signaling pathway (Figure 3). For instance, starting from the genes that encode proteins responsible for uptake and perception of QS peptides, we measured *LuxPQ*, *LuxN,* and *CqsS* transcripts’ abundance. The protein complex LuxPQ is responsible for sensing AI-1 peptide and consists of two protein domains transcribed by *LuxP* and *LuxQ* genes in *V. alginolyticus*. We observed a more than twofold statistically significant downregulation of *LuxP* domain in both phage-resistant mutants, while *LuxQ* domain did not exhibit any statistically significant change. Protein transcribed from the *LuxN* gene is responsible for sensing AI-2 peptide. In our study, we observed a statistically significant 39-fold and 80-fold depletion in *LuxN* transcript levels for the VaphiSt2- and VaAthena1-resistant strains, respectively, when compared to the phage-susceptible strain. Regarding *CqsS*, which encodes a protein responsible for the perception of the CA-1 peptide, we observed a statistically significant fourfold downregulation in VaAthena1-resistant strains, while VaphiSt2 remained unaffected. The transcription of QS peptide biosynthesis genes is linked to the corresponding QS peptide abundance in the cytoplasm. *LuxM*, *LuxS*, and *CqsA* are three genes responsible for the biosynthesis of QS peptides (AI-1, AI-2, and CA-1, respectively) in *V. alginolyticus*. Our results showed a statistically significant downregulation of *LuxM*, with a 2-fold decrease in the VaphiSt2-resistant strain and a 2.5-fold decrease in the VaAthena1-resistant strain. *LuxS* transcript abundance exhibited a statistically significant downregulation of approximately threefold only in the case of VaphiSt2 mutants. One of the two isoforms of *CqsA*, annotated in the respective *V. alginolyticus* strain, showed a mild downregulation pattern in both resistant strains, and the second one showed a statistically significant 2-fold downregulation in VaAthena1-resistant strains. These findings suggest that the development of phage resistance in *V. alginolyticus* can profoundly impact the perception and biosynthesis of specific QS peptides.

**Figure 4:**
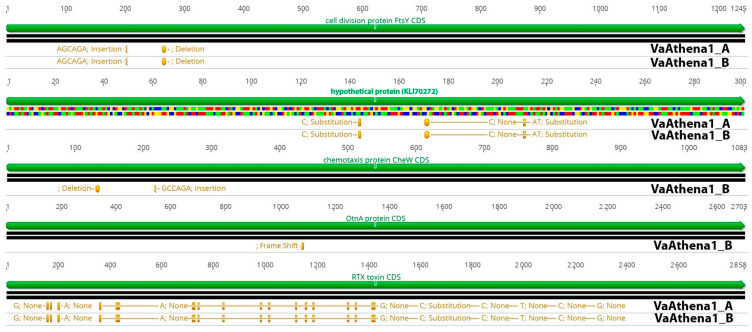
Comparison of five CDSs exhibiting SNPs in *V. alginolyticus*-resistant strains VaAthena1. Green color represents CDS and orange represents SNP positions and information about protein effect on the protein in the resistant bacteria.

The abundance of QS peptides is responsible for the transcription of the phosphokinase *LuxU* in vibrios. In our study, we observed a statistically significant downregulation of *LuxU*, with a 12-fold decrease in the VaphiSt2-resistant strain and a 50-fold decrease in the VaAthena1-resistant strain. *LuxU* is involved in the transcriptional regulation of the *LuxO* gene, for which two isoforms have been annotated in *V. alginolyticus* V1 strain. The transcriptional study revealed that one isoform of the *LuxO* gene was statistically significantly downregulated in both resistant strains. The second isoform of *LuxO* remained statistically downregulated in the case of VaphiSt2-resistant strains, while it showed a statistically significant 3.2-fold upregulation in the case of VaAthena1. *LuxO* is responsible for regulating the switch between *LuxR* and *aphA* genes, which are the master regulators controlling approximately 400 genes [31]. Regarding *LuxR*, we observed a complete depletion of this gene’s expression with statistically significant downregulation of 24-fold and 150-fold in VaphiSt2 and VaAthena1-resistant strains, respectively. *aphA* was statistically significantly downregulated by 2.0-fold in VaphiSt2, and by at least 12-fold in VaAthena1-resistant strains. The value of the aphA/LuxR transcript ratio in wild-type cells indicated an LCD state, with a ratio of approximately three. In the bacteriophage-resistant mutants, the relative transcriptional ratio of aphA/LuxR increased to approximately 36 and 38 for the VaphiSt2 and VaAthena1-resistant strains, respectively (Figure 4), potentially showing a locked LCD status. Additionally, a chaperone transcribed from *Hfq* is responsible for the natural conformation of LuxR and aphA proteins. Chaperone *Hfq* was downregulated by a statistically significant manner of 2.5-fold in VaAthena1-resistant strains only.

### 3.3. Transcriptional Regulation of QS-Related Genes

*LuxR* and *aphA* act as master regulators of many genes associated with bacterial fitness. Therefore, we investigated the transcriptional status of membrane, biofilm, and virulence-related genes of *V. alginolyticus* known to be regulated by QS activity. Starting with the secreted protein *Wza*, which encodes for an exopolysaccharide secretion protein, we observed a 20-fold downregulation and a nearly complete depletion in resistant strains VaphiSt2 and VaAthena1, respectively. The *trh* gene, which encodes for a thermostable direct hemolysin (TDH-related hemolysin), was downregulated by 4-fold and at least by 6-fold in the resistant strains VaphiSt2 and VaAthena1, respectively, without being statistically significant. The anthranilate synthase encoded by *trpE* showed a statistical significance downregulation of 3.5-fold and 2-fold in resistant strains VaphiSt2 and VaAthena1, respectively. The ferric uptake regulator encoded by the *fur* gene exhibited statistically significant downregulation of 1.5-fold and 3.0-fold in resistant strains VaphiSt2 and VaAthena1, respectively.

Regarding the major facilitator superfamily transporters-related genes (MFS), the *Uhpc* gene, responsible for sensing glucose-6-phosphate in the periplasmic space, exhibited a significant downregulation in the resistant strains. We observed a 25-fold decrease in the VaphiSt2-resistant strain and a 45-fold decrease in the VaAthena1-resistant strain, both statistically significant. Furthermore, the efflux pump Bcr/CflA showed a substantial depletion in both resistant strains. Specifically, we observed a significant 220-fold decrease in the VaphiSt2 strain and a 70-fold decrease in the VaAthena1 strain. The transcriptional study of QS cassette and QS regulated genes shows that bacteriophage-resistant strains undergo a transcriptional downregulation, after the interaction with lytic bacteriophages.

### 3.4. Comparative Genomic Analysis of Phage-Resistant Strains

Genome mutations on transcriptional regulators could be responsible for altering the transcriptional status of some genes in phage-resistant mutants; thus, we also examined possible single nucleotide polymorphisms (SNPs) instigated after the development of phage resistance (Tables S3 and S4; Figure 4). For that reason, we sequenced the genome of two phage-resistant colonies resistant to bacteriophage Athena1, which were used in the virulence assay and were found to possess a lower virulence capacity (Figure 1C). By using a threshold of 25% in variance presence, we were able to detect in total, 366 and 279 polymorphisms compared to the wild-type strain. 6,104,378 reads for VaAthena1_A and 7,768,406 reads for VaAthena1_B were utilized with an average read length of 250 bps. This corresponded to at least 300X sequence depth. Common mutations shared between both resistant strains of VaAthena1 were the cell division protein encoded by *FtsY* gene, SNPs on RTX toxin, and SNPs on a nucleoside permease. More specifically for both VaAthena1 mutants a deletion of six nucleotides at position 264 nt was observed on *FtsY* responsible for transcribing a cell division protein (KLI70360), a mutation which removed one Glutamate and one Alanine amino acids. In addition to the previously mentioned polymorphisms, we identified a second mutation in the sequenced colonies. This mutation involved the insertion of six nucleotides at position 204, resulting in the addition of one Alanine (Ala) and one Glutamate (Glu) amino acid residue. These mutations are in a tandem repeat motif of the amino acid sequence of FtsY cell division protein. Interestingly in both resistant strains, three mutations were observed to a hypothetical protein (KLI70272), leading to two amino acid substitutions. Specifically, at position 144 nucleotide (nt), Glutamate (Glu) was substituted with Aspartate (Asp), while at position 171 nt, Histidine (His) was replaced with Tyrosine (Tyr). The third mutation was silent and did not result in any protein change. Furthermore, VaAthena1_B exhibited two additional noteworthy mutations in the chemotaxis-secreted protein CheW (KLI71480). Specifically, a deletion at position 131 nt resulted in the removal of one Glutamate (Glu) and one Proline (Pro) amino acid residue. In addition, an insertion at position 219 nt led to the addition of one Proline and one Glutamate amino acid residue. One nt deletion in *OtnA* gene (KLI70626), related to a polysaccharide secrete protein, resulted in a frameshift at position 1087, which could potentially impair its function Finally, the RTX toxin (KLI69968) and three ribosomal RNAs (5 s, 23 s, and 16 s) were identified as mutual targets of multiple single nucleotide polymorphisms (SNPs) when compared to the wild-type strain. Overall, these results showed that the bacteriophage Athena1 induced a lower number of SNPs in *V. alginolyticu*s-resistant strains compared to bacteriophage *φ*St2, which harbored almost double SNPs (~500), as recently described [9]. Additionally, resistant mutants to φSt2 introduce SNPs to well-known transcriptional regulators, a phenomenon not observed in resistant mutants to Athena1 lytic phage. Although the cellular receptors are unknown for lytic bacteriophage Athena1, no mutations were detected in previously identified phage adsorption sites of Caudoviricetes order, such as OmpF, BtuB, LamB, and TolC proteins, in both resistant to Athena1 mutants [32].

## 4. Discussion

The role of the quorum-sensing (QS) cascade in aquatic species, particularly vibrios, has been extensively documented as a fundamental mechanism for survival under diverse abiotic stresses, nutrient availability, variable environmental conditions, and population cell density [33,34,35,36]. Additionally, strain-specific responses have been observed in different environments [37]. Recent studies have highlighted the connection between phage invasion and the behavioral response regulated by QS [38]. There is a growing body of research evidence demonstrating the interplay between QS regulation and behavioral traits in vibrios. However, the biological significance and the relationship between QS and the acquisition of bacteriophage resistance remains unclear. In this study, our aim was to investigate, for the first time, the expression patterns of the QS cassette and QS-related genes in *V. alginolyticus* and monitor newly introduced phenotypic traits of *V. alginolyticus* under low-cell density conditions, following interactions with two distinct lytic bacteriophages.

### 4.1. Instigated Mutations during the Development of Acquired Phage Resistance Could Potentially Affect QS Regulation and V. alginolyticus Physiology

Generation of bacteriophage-resistant mutants under a long phage exposure in marine Gram-negative bacterial species, such as *Flavobacterium psychrophilum* [39] and *Vibrio anguillarum* [40], can select nucleotide polymorphisms (SNPs), insertions, or deletions in phage adsorption proteins, which can result in a permanent bacteriophage resistance state. On the other hand, previous studies on sequenced phage-resistant mutants during short-term exposure to lytic bacteriophages have shown that vibrios can also introduce SNPs in transcriptional regulators, such as sigma factors and histidine kinase systems [41]. These mutations could potentially lead to transcriptional alterations in numerous genes, possibly also including QS cassette. In this study, we present results from two independent phage-resistant mutants for the lytic bacteriophage Athena1, comparing them to previously identified and characterized phage-resistant mutants for the large genome-sized bacteriophage *φ*St2 [9], both exhibiting a myovirus morphotype. In comparison, bacteriophage *φ*St2 imposed the bacterial cells to approximately ~35% more SNPs than Athena1 and possibly to a more severe host membrane remodel [42]. Differences in bacteriophage genome sizes are indicative of the availability of various molecular tools that allow the efficient host hijacking under different environmental and/or cellular conditions. Consequently, bacteriophages harboring larger genomes may be able to result in a higher number of SNPs into the population and subject the host to a more intense metabolic reprogramming, possibly conferring a resistant phenotype to other bacteriophages as documented here. Previously, we reported the presence of mutations in transcriptional regulators of resistant mutants of *φ*St2, such as the MerR family transcriptional regulator and the *UhpA* transcriptional regulator (amino acid substitution), as well as RpoD (sigma factor 70). These mutations could potentially affect the simultaneous transcriptional status of multiple pathways involved in QS cassette, uptake of 6P-glucose, biofilm formation capacity, and virulence [43,44,45,46]. On the other hand, resistant mutants to lytic bacteriophage, Athena1, did not exhibit SNPs in transcriptional regulators or known phage attachment proteins. Results revealed SNPs in tandem repeats of CheW and FtsY proteins. Transposition of tandem repeat motifs in proteins could affect protein function [47]. Malformation of Chew protein could alter bacterial chemotaxis state and subsequently virulent capacity [48]. Similarly, FtsY cell division protein tandem repeat translocation could impact its function and subsequently affect normal cell physiology [49]. Interestingly, one resistant mutant to Athena1 bacteriophage also introduced a frameshift to OtnA protein, which majorly impacts protein translation. OtnA protein is associated with capsule coherence in *Vibrio cholerae* [50]. Impaired capsule synthesis in Gram-negative bacteria is associated with phage resistance in *Klebsiella pneumoniae*, when phages target capsule-associated adsorption sites [51].

Furthermore, the RTX toxin of *V. alginolyticus* exhibited a significant number of SNPs in both resistant mutants. SNPs in RTX toxins can attenuate their cytotoxicity and subsequently lead to differences in virulence capacity [52]. The deletion of a Zot-like toxin was reported in phage-resistant mutants of *Vibrio anguillarum* by Leon et al. [41], which also resulted in reduced virulence in those strains. These findings suggest that these proteins are frequently targeted by SNPs in *Vibrio* species and could be associated with a reduced virulent phenotype.

### 4.2. QS Is Perturbed in V. alginolyticus upon the Development of Phage Resistance

While cell density typically governs quorum-sensing (QS) regulation among bacterial populations, there are numerous reports highlighting QS regulation under various environmental niches [53]. These diverse regulatory mechanisms contribute to the emergence of different phenotypic traits within a population and operate at the transcriptional, post-transcriptional, and post-translational levels of regulatory proteins [54].

In this study, we clearly observed a transcriptional shift in the QS peptide sensing pathway within a bacterial population experiencing a biotic stress. We found that the transcriptional status of the two-component sensor systems related to autoinducers AI-1 and AI-2 (*LuxP*, *LuxQ*, *LuxN*) was downregulated in both phage-resistant strains. Additionally, in the resistant strains to Athena1, the CA-1 histidine kinase-sensing system (*CqsS*) also appeared depleted. All these QS two-component systems are located in the inner bacterial membrane and can sense QS peptides presence and abundance in the periplasm [55,56].

It is reasonable to hypothesize that malfunctioning or reduced transcription of the QS peptide-sensing system could lead to a false perception of population density, resulting in perturbation of the QS cascade system. For example, reduced sensing of AI-2 by *LuxQ* can have detrimental effects on the transcriptional status of downstream QS factors, such as *LuxU* and the regulator *LuxO* [57]. Similarly, decreased *LuxN* sensing capacity, regulated by AI-1 abundance, can also affect *LuxO* phosphorylation [58,59]. In the case of strains resistant to Athena1, diminished expression of the *CqsS*-sensing protein is known to impact the activity and transcription of the *LuxO* regulator. The exact reasons for the reduced transcriptional status of genes encoding sensing proteins are yet to be determined. However, vibrios possess a well-orchestrated regulatory network of the QS cascade under various conditions [54], suggesting that the reduced transcription may be a collateral event during the complex metabolic reprogramming that occurs [9].

Corresponding peptide synthases *LuxM* (AI-1), *LuxS* (AI-2), and *CqsA* (CA-1) were also found to have reduced transcription in both resistant strains. Potentially decreased QS peptide synthesis can have an immediate effect on sensing and subsequently impact the phosphokinase ability of sensors *LuxQP* and *LuxN* under low-cell density conditions [60]. Similarly, the *CqsA* synthase, responsible for an intergenus communicator peptide, CA-1 [55], showed reduced transcription. These findings possibly indicate a perturbed sensing of QS peptides and presumably QS peptide production during the low-cell density state of the population after the development of acquired phage resistance.

The decreased transcript levels of all histidine kinases suggests a reduced level of phosphorylation on *LuxU* phosphotransfer, subsequently affecting the phosphorylation state of the critical *LuxO* regulator. The relative transcript accumulation of *LuxU* was also significantly decreased in both resistant strains. An impaired perception of LCD metabolic state could even more reduce the phosphorylation of the *LuxO* regulator. Notably, *V. alginolyticus* strain V1 was found to possess two isoforms of the *LuxO* gene. We observed that *LuxO* was altered significantly in terms of transcription, although in the case of VaAthena1-resistant mutants, one isoform appeared upregulated, possibly compensating for the total transcriptional status. This indicates that the *LuxO* regulator independent from its transcriptional state, might exist in a high dephosphorylated state [58].

Based on the information obtained so far, we decided to investigate the transcription of two key regulators, *aphA* and *LuxR*, which play a crucial role in the low cell-density (LCD) and high-cell density (HCD) states interplay, respectively, and are involved in the transcription of over 200 genes [61]. It is well established that intracellular abundance of autoinducers activates *LuxR*, the master regulator responsible for repressing virulence and biofilm formation, while the absence or reduced sensing of autoinducers activates *aphA*, the master regulator responsible for inducing both virulence and biofilm formation [17]. In our study, the perturbed sensing and production of autoinducers resulted in a significant depletion of *LuxR* regulator transcription, while the transcription of *aphA* was also downregulated compared to the wild-type phage-susceptible strains.

Due to the reciprocal interplay between these regulators, acting as both activators and repressors, their transcriptional status and ratio can provide insights into the cell density state [62]. The relative transcript ratio of *aphA/LuxR* (Figure 3) showed a sustained and locked LCD metabolic state with limited sensing and intracellular abundance of autoinducers [17]. In the case of VaAthena1-resistant strains, the chaperone Hfq, responsible for *aphA* conformation, was significantly depleted as well, indicating that not only *aphA* transcription is reduced but also protein misfolding may be present. This can affect the normal function of *aphA* and the sensing capacity of autoinducers, as shown by Lenz et al. [18].

Overall, these results demonstrate an irregular function of the QS cascade at both the transcriptional and potentially post-translational levels during the LCD state, which accounts for various phenotypic traits observed in *V. alginolyticus* upon phage infection.

### 4.3. QS Cascade Malfunction Due to the Developemtn of Phage-Resistance Can Be Evident for Diverse Phenotypic Traits

The impairment of genes involved in the QS cascade could directly affect phenotypic traits. *aphA*, which has been described as a major regulator of natural competence in *Vibrio cholerae* [63], also plays a similar role in *Vibrio alginolyticus* after the acquisition of phage resistance. The transcriptional status of many QS-related genes, such as *LuxQ* [57], *LuxU* [64], and *LuxO* [65], has been correlated with fitness costs under various ecological habitats. Furthermore, in addition to studying the transcriptional interplay of *aphA* and *LuxR* genes in populations with acquired bacteriophage resistance, we investigated the transcriptional levels of genes involved in virulence and controlled by *aphA*.

We found that the polysaccharide export protein gene, *Wza*, was depleted in both resistant strains. In Gram-negative species, the *Wza* gene is known to be involved in virulence, and its impairment can result in a less-virulent phenotype [66]. The anthranilate synthase gene, *trpE*, was also found to be depleted in the phage-resistant strains and has been associated with virulence phenotype. Inhibition of *trpE* can terminate virulence in *Streptococcus pneumoniae* [67]. Additionally, the thermostable direct hemolysin gene, *trh*, which is a major virulence gene in *Vibrio parahaemolyticus* [68], was downregulated in both resistant strains. The ferric uptake regulator gene, *fur*, which is an important regulator and controller of virulence in *Vibrio* species [69], was also found to be downregulated. Furthermore, we report for the first time a major facilitator superfamily gene, *Bcr/CflA*, which is a transporter responsible for virulence [70], and it was dramatically depleted in both resistant mutants. Interestingly, overexpression of efflux pumps has been linked to the development of multidrug-resistant Gram-negative bacteria [71].

The glucose-6-phosphate-sensing protein encoded by *Uhpc* was found to be downregulated in both resistant strains, leading to reduced sensing and potential uptake of phosphorylated glucose by the cells. This phenomenon has been linked in the past with a reduced biofilm formation capacity [72]. These findings corroborate here with the reduced virulent phenotype observed in the resistant strains against both lytic bacteriophages and the reduced biofilm capacity in the VaAthena1-resistant strains. In the case of increased biofilm formation capacity in the VaphiSt2-resistant strain, we also observed a reduced growth rate during the planktonic phase. It is possible that the VaphiSt2-resistant mutants decrease their potent growth rate in favor of biofilm formation. Biofilm formation capacity could also be fueled by unknown autoinducer peptides [73]. Interestingly, *LuxS* transcripts, which were significantly reduced only in these phage-resistant strains, corroborate the induced biofilm formation capacity and reduced virulence, as also observed in *V. alginolyticus* by Ye et al. [74] and in *Actinobacillus pleuropneumoniae* by Li et al. [75] in *LuxS*-impaired mutants.

**Figure 5:**
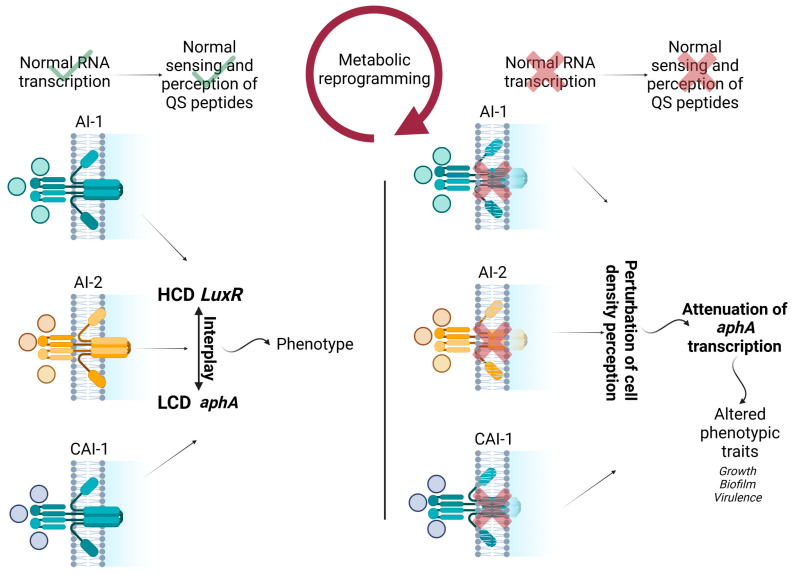
Schematic representation of the interplay between transcript abundance of QS membrane proteins, QS biosynthesis genes, metabolic reprogramming, and observed phenotypic alterations in the study. HCD represents high cell density, and LCD represents low cell density. Wild type strain functions are shown with check marks. Perturbed functions of bacteriophage resistant strains are shown with cross marks.

Overall, our results report a potential association between QS transcriptional fate and the emergence of new phenotypes, which may result from a fitness cost during the metabolic adaptation strategy of bacterial population dynamics (Figure 5) [44].

## 5. Conclusions

Studying bacteriophage-resistant strains, both in vitro and in the context of bacteriophage biocontrol, is of utmost importance for monitoring and correlating fitness alterations. Vibrios, in particular, exhibit unique biochemical plasticity compared to other Gram-negative marine bacteria, allowing them to overcome or avoid viral infections. This plasticity could be imposed by genomic liaisons and/or a global transcriptional reprogramming. An orchestrated phage-specific metabolic reprogramming has emerged as a potential phage evasion strategy [9], in which the QS cascade could play a pivotal role as seen here. It is documented that phages tend to select for QS-proficient bacteria in many species [38]. A transcriptionally affected QS cassette is monitored here with key genes such as *LuxS*, *LuxU*, *LuxR,* and *aphA* being significantly downregulated presenting a perturbed cascade, after developing phage-resistance. Also, the ability of QS to regulate a large number of genes in response to interaction with lytic bacteriophages suggests that a transcriptional adaptation strategy could explain a significant portion of the fitness variations that phage-resistant marine vibrios develop, such as biofilm formation capacity, growth, and virulence. Manipulating the QS cascade of multidrug-resistant Gram-negative bacteria [76] and bacteria with acquired phage resistance holds promise for developing more efficient strategies against bacterial infections. Future experiments studying the SNPs presented here and with targeted mutants on genes, in which their relative transcript levels appeared depleted, will enhance our knowledge surrounding the contribution of QS on acquired phage resistance.

## Figures and Tables

**Table 1:** Main characteristics of the phages used in this study.

Phage	Morphotype	Class	Lifestyle	Genome (nts)	Predicted ORFs	Latent Period/Burst Size	Resistant Bacterial Strain	Accession Number	Reference
*φSt2*	myovirus	*Caudoviricetes*	Lytic	250.485	412	30 min/ 97 pfu/cell	VaphiSt2	KT919973	[20,22]
*Athena1*	myovirus	*Caudoviricetes*	Lytic	39.826	57	30 min/ 70 pfu/cell	VaAthena1	MG640035	[21]

Abbreviations: ORFs: open reading frame.

**Table 2:** Cross-infection test (− for the absence of lytic plaques; + for high lytic activity) of wild-type and resistant strains against the lytic bacteriophages used in the present study.

*Vibrio alginolyticus* Strains	φSt2	Athena1
Wild-type (Control)	+	+
VaphiSt2	−	−
VaAthena1	+	−

## Data Availability

The *V. alginolyticus* strain V1 genome is available in the GenBank public DNA repository under the accession number LCUM00000000. The genomes of the bacteriophage-resistant strains of bacteriophage *φ*St2, which are partially included in the analysis here have been deposited in GenBank during conducting a previous work [9] under the accession numbers for VaphiSt2_A, JAGFOI000000000 and for *V. alginolyticus* strain VaphiSt2_B, JAGFOL000000000. In regard to phage-resistant strains to lytic bacteriophage, Athena1, sequenced strains have been deposited n Genbank under the accession numbers for VaAthena1_A JAUALG000000000 and for VaAthena1_B JAUALF000000000.

## Supplementary Materials

The following supporting information can be downloaded at: https://www.mdpi.com/article/10.3390/microorganisms11092273/s1, Supplemental Table S1. Primers used in the study. Supplemental Table S2. Relative transcript abundances of genes involved in the study. Supplemental Table S3. SNPs corresponding to the bacteriophage resistant strain VaAthena1_A, Supplemental Table S4. SNPs corresponding to the bacteriophage resistant strain VaAthena1_B.
