# Supplementary material for: Perturbation of Quorum Sensing after the Acquisition of Bacteriophage Resistance Could Contribute to Novel Traits in Vibrio alginolyticus
Source: Microorganisms. 2023 Sep 10;11(9):2273. doi: 10.3390/microorganisms11092273 (PMC10535087; doi:10.3390/microorganisms11092273)
Supplement: Supplementary file 1 [file microorganisms-11-02273-s001.zip › Table S1. Primers list of qPCR platform.pdf]

Skliros et al., 2023. Table S1. Primers list with average reaction efficiency (E) taken from slopes and r^2 of rt-qPCR platform. Primers list, which were used to study relative transcript levels of *Vibrio alginolyticus* strain V1 genes involved in QS (A) and QS regulated genes (B).

A.

**Primers amplifying genes of QS cassette**		**Average Reaction Efficiency**	$\mathbf{r^2}$
Gene	Primers (R’/F’)		
LuxQ	Reverse-5’-TGGCGTGCCTAGTTTTAGCT-3’<br>Forward-5’-CCAACTTCCGCATCTTGGTG -3’	1.976	0.999
LuxP	Reverse-5’-CAACGGCCAATTCACGACTT -3’<br>Forward-5’-TGGGAACATCATCAGCCGTT -3’	1.979	0.999
LuxM	Reverse-5’-GTTGATGCCAGCTTTGCACT -3’<br>Forward-5’-GCCCTCTTGCTCAAATCGTC -3’	1.985	0.999
CqsA1	Reverse-5’-CACGTAGAGGAGCGATGGTC-3'<br>Forward-5’-CGTCACGGTTCAGGGATCAT-3'	1.995	0.999
CqsA2	Reverse-5'-TGTTCCTTCCAGTTGCAGGG-3'<br>Forward-5'-GTCCGCGACTTTCTCGAAGA-3’	1.985	0.999
Hfq	Reverse-5’-TCACCTTGTGGACGTTCACC -3’<br>Forward-5’-AGCACGCGATCTCAACAGTT -3’	1.954	0.999
LuxO1	Reverse-5’-ATCCGTGATAGTTGTGGCGA-3’<br>Forward-5’-AAACGCGCCAAATTGGAAGA-3’	1.978	0.999
LuxO2	Reverse-5’-CGACGGACTGACATCAAGGT-3’<br>Forward-5’- CGAGCAAGCGATTGAAGCAT-3’	1.990	0.999
LuxS	Reverse-5’-ACCGAAACACCAGCATCCAA-3’<br>Forward-5’- CGGCAGCAATGCACTCTTTA-3’	1.996	0.999
LuxU	Reverse-5’-TGGACATCGCCAGTTCACAA-3’<br>Forward-5’- ATCAGCCACGCGTTGAAAAG-3’	1.993	0.999
CqsS	Reverse-5’-CACGTAGAGGAGCGATGGTC-3'<br>Forward-5’-CGTCACGGTTCAGGGATCAT-3'	1.980	0.999
aphA	Reverse- 5'-ACCACTCTCCTAAAGCGCTG-3'<br>Forward-5'-CAAGAAGGCAAACCAGACCG-3'	1.986	0.999
LuxR	Reverse-5’- TGTTGAAGCACTCCACTCGA -3’<br>Forward-5’- TGCGATGATCGAGCTTGTCA -3’	1.991	0.999
LuxN	Reverse-5’AAAATGCCACCAACAGAGCC-3’<br>Forward-5’TTTGGATAAGCAGCAACGCC-3’	1.996	0.999

B.

**Primers amplifying genes of QS regulated genes**		**Reaction Efficiency**	**R2**
Gene	Primers (R'/F')		
UhpC	Reverse-5'-CCGCAACCAATGAACCAACA-3'	1.991	0.999
	Forward-5'-AGCCTGATTAATGCCAACGC -3'		
trh	Reverse-5'-TTACCTGCGGTAAAGCCAGG-3'	1.989	0.999
	Forward-5'-CGCAAAACTTCCGCCAAAAC-3'		
trpE	Reverse-5'-CTAGCGAGTGGTAACGTGCA-3'	1.975	0.999
	Forward-5'-GAGCACCAAAACCATGCGAT-3'		
Bcr/Cfla	Reverse-5'- 5'-GCTCGTCTGGTACTAGCGTT-3'	1.996	0.999
	Forward-5'-TCGGGGATTGCCAAAGATCT -3'		
fur	Reverse-5'-GTCTGGCTGCTGAAGTACTT-3'	1.997	0.999
	Forward-5'-GCTGAAGGATGCGGGTTTAA -3'		
Wza	Reverse-5'-CACCATAGACCTGCACGGAA -3'	1.990	0.999
	Forward-5'- AACGCTGACTCAAACGAGGA-3'		
