# Supplementary material for: Perturbation of Quorum Sensing after the Acquisition of Bacteriophage Resistance Could Contribute to Novel Traits in Vibrio alginolyticus
Source: Microorganisms. 2023 Sep 10;11(9):2273. doi: 10.3390/microorganisms11092273 (PMC10535087; doi:10.3390/microorganisms11092273)
Supplement: Supplementary file 1 [file microorganisms-11-02273-s001.zip › Table S2. Relative transcript abudances of phage resistant mutants.pdf]

Skliros et al., 2023. Table S2. Average relative transcript abundances (±SE) of *Vibrio alginolyticus* strain V1 genes and its respective resistant strains to bacteriophages *φ*St2 (VaphiSt2) and Athena1 (VaAthena1). **A** for QS cassette genes, **B** for genes regulated by QS cascade. Heat maps that appear in Figure 3 of main text correspond to the presented colored values here. *P* value represents t-tests between Wild type and respective resistant strain. All treatments passed normality t-test. If equal variance test failed a Welch's test was applied. Statistical significance ($P \leq 0.05$) is represented by bold *P* value.

A.

**Relative expression levels of QS cassette genes**															
	LuxQ	**±SE**	***P***	***LuxM***	***±SE***	***P***	***Hfq***	***±SE***	***P***	***LuxU***	***±SE***	***P***	***aphA***	***±SE***	***P***
**Wild type**	0.072	0.014	na	0.120	0.003	na	1.048	0.080	na	0.289	0.011	na	3.633	0.039	na
**VaphiSt2**	0.059	0.003	0.509	0.057	0.010	**0.007**	0.746	0.154	0.230	0.024	0.005	**0.000**	1.618	0.116	**0.002**
**VaAthena1**	0.015	0.000	0.077	0.046	0.011	**0.006**	0.407	0.108	**0.018**	0.006	0.001	**0.000**	0.306	0.015	**0.000**
	LuxP	**±SE**	***P***	***CqsA1***	**±SE**	***P***	***LuxO1***	**±SE**	***P***	***LuxO2***	**±SE**	***P***	***LuxN***	**±SE**	***P***
**Wild type**	0.438	0.005	na	0.371	0.164	na	0.016	0.001	na	0.067	0.008	na	1.284	0.131	na
**VaphiSt2**	0.065	0.000	**0.034**	0.002	0.000	0.208	0.006	0.001	**0.011**	0.012	0.000	**0.032**	0.032	0.006	**0.016**
**VaAthena1**	0.088	0.001	**0.038**	0.004	0.000	0.209	0.051	0.007	**0.050**	0.006	0.001	**0.024**	0.016	0.003	**0.016**
	LuxR	**±SE**	***P***	***LuxS***	**±SE**	***P***	***CqsA2***	**±SE**	***P***	***CqsS***	**±SE**	***P***			
**Wild type**	1.233	0.135	na	0.753	0.108	na	0.036	0.001	na	0.112	0.006	na			
**VaphiSt2**	0.045	0.009	**0.018**	0.269	0.003	**0.021**	0.031	0.002	0.262	0.117	0.008	0.694			
**VaAthena1**	0.008	0.004	**0.018**	0.579	0.057	0.308	0.016	0.002	**0.002**	0.027	0.005	**0.000**			

B.

Relative expression levels of QS cascade regulated genes									
	Uhpc	**±SE**	***P***	***trh***	***±SE***	***P***	***trpE***	***±SE***	***P***
**Wild type**	0.117	0.050	na	0.194	0.047	na	0.116	0.010	na
**VaphiSt2**	0.004	0.000	**0.04**	0.052	0.006	0.102	0.034	0.015	**0.050**
**VaAthena1**	0.002	0.000	**0.03**	0.031	0.005	0.127	0.059	0.014	**0.023**
	fur	**±SE**	***P***	***Wza***	***±SE***	***P***	***Bcr/Cfla***	***±SE***	***P***
**Wild type**	0.783	0.030	na	0.023	0.001	na	4.270	1.057	na
**VaphiSt2**	0.642	0.017	**0.040**	0.001	0.000	**0.006**	0.059	0.004	**0.031**
**VaAthena1**	0.245	0.042	**0.002**	0.000	0.000	**0.005**	0.020	0.009	**0.030**
