# Supplementary material for: Perturbation of Quorum Sensing after the Acquisition of Bacteriophage Resistance Could Contribute to Novel Traits in Vibrio alginolyticus
Source: Microorganisms. 2023 Sep 10;11(9):2273. doi: 10.3390/microorganisms11092273 (PMC10535087; doi:10.3390/microorganisms11092273)
Supplement: Supplementary file 1 [file microorganisms-11-02273-s001.zip › Table S3. SNPs corresponding to the bacteriophage resistant strain VaAthena1_A.pdf]

Skliros et al., 2023. Supplemental Table S3. Single Nucleotide Polymorphisms of resistant strain **VaAthena1_A** for each Contig separately. Tables show Nucleotide shift, position, change, polymorphism, type, variant frequency and in the case of a mutation in a CDS product, protein, effect and protein_id.

Contig 2

Name	Position	Length	Change	Polymorphism Type	Variant Frequency	Amino Acid Change	CDS	product	Protein Effect	protein_id
A	152043	1	C -> A	SNP (transversion)	40.00%	K -> N	diguanylate cyclase CDS	diguanylate cyclase	Substitution	KLI73954.1
T	222636	1	G -> T	SNP (transversion)	28.60%	G -> V	hypothetical protein CDS	hypothetical protein	Substitution	KLI74018.1

Contig 5

Name	Minimum	Maximum	Length	Change	Coverage	Polymorphism Type	Variant Frequency
G	22	22	1	T -> G	91	SNP (transversion)	38.50%
A	35	35	1	G -> A	68	SNP (transition)	32.40%
GA	37	38	2	TT -> GA	51 -> 67	Substitution	29.9% -> 31.4%
T	53	53	1	C -> T	32	SNP (transition)	31.30%
G	272000	272000	1	A -> G	220	SNP (transition)	25.00%
C	272002	272002	1	T -> C	220	SNP (transition)	25.50%
C	272005	272005	1	G -> C	225	SNP (transversion)	26.20%
A	272009	272009	1	T -> A	232	SNP (transversion)	27.20%
AAG	272013	272012	0		236	Insertion	25.00%
TT	272016	272017	2	AA -> TT	246 -> 247	Substitution	27.5% -> 27.6%
A	272019	272019	1	G -> A	252	SNP (transition)	28.20%
G	272023	272023	1	A -> G	262	SNP (transition)	28.20%
G	272025	272025	1	C -> G	263	SNP (transversion)	30.00%
C	272027	272027	1	G -> C	264	SNP (transversion)	30.70%
CG	272032	272031	0		261	Insertion	30.70%
G	272035	272035	1	C -> G	266	SNP (transversion)	30.80%
C	272037	272037	1	G -> C	267	SNP (transversion)	30.70%

Contig 6

Name	Minimum	Maximum	Length	Change	Coverage	Polymorphism Type	Variant Frequency
AT	49	50	2	TC -> AT	194 -> 195	Substitution	34.9% -> 35.1%
T	52	52	1	C -> T	202	SNP (transition)	34.70%
T	64	64	1	A -> T	223	SNP (transversion)	41.30%
T	152	151	0		223	Insertion	55.60%
G	155	155	1	A -> G	223	SNP (transition)	56.50%
TCA	157	159	3	ATT -> TCA	213 -> 223	Substitution	56.1% -> 58.7%
GT	162	163	2	TC -> GT	227 -> 228	Substitution	55.3% -> 55.5%
TTAAAATGGCTACGTAGCTCAGCTGGTTAGAGCACATCACTCATAATGATGGGGTCACAGGTTCGAATCCCGTCGTAGCCACCAT	165	165	1	A -> TTAAAATGGCTACGTAGCTCAGCTGGTTAGAGCACATCACTCATAATGATGGGGTCACAGGTTCGAATCCCGTCGTAGCCACCAT	164 -> 230	Insertion	34.1% -> 51.3%
A	166	165	0		161	Insertion	35.40%
TT	166	165	0		149 -> 151	Insertion	30.2% -> 31.1%
T	166	165	0		148	Insertion	29.70%
T	300	300	1	G -> T	139	SNP (transversion)	32.40%
AA	310	311	2	CC -> AA	173 -> 222	Substitution	42.2% -> 56.3%
T	390	390	1	C -> T	277	SNP (transition)	44.80%
C	420	420	1	T -> C	369	SNP (transition)	36.60%
A	428	428	1	T -> A	410	SNP (transversion)	36.60%
A	436	436	1	T -> A	486	SNP (transversion)	47.30%
C	437	437	1	T -> C	487	SNP (transition)	37.80%
G	191752	191752	1	A -> G	296	SNP (transition)	28.00%

Contig 7

Name	Minimum	Maximum	Length	Change	Coverage	Polymorphism Type	Variant Frequency	Amino Acid Change	CDS	product	Protein Effect	protein_id
C	216879	216879	1	T -> C	250	SNP (transition)	30.80%		calcium-binding protein CDS	calcium-binding protein	None	KLI72868.1
C	216885	216885	1	A -> C	252	SNP (transversion)	31.00%		calcium-binding protein CDS	calcium-binding protein	None	KLI72868.1
C	216888	216888	1	G -> C	254	SNP (transversion)	30.70%		calcium-binding protein CDS	calcium-binding protein	None	KLI72868.1
T	216921	216921	1	C -> T	266	SNP (transition)	30.80%		calcium-binding protein CDS	calcium-binding protein	None	KLI72868.1
T	216924	216924	1	C -> T	262	SNP (transition)	30.50%		calcium-binding protein CDS	calcium-binding protein	None	KLI72868.1
C	216927	216927	1	G -> C	261	SNP (transversion)	29.90%		calcium-binding protein CDS	calcium-binding protein	None	KLI72868.1
A	216942	216942	1	T -> A	254	SNP (transversion)	27.20%		calcium-binding protein CDS	calcium-binding protein	None	KLI72868.1

Contig 13

Name	Minimum	Maximum	Length	Change	Coverage	Polymorphism Type	Variant Frequency
A	286321	286321	1	G -> A	290	SNP (transition)	27.90%

Contig 14

Name	Minimum	Maximum	Length	Change	Coverage	Polymorphism Type	Variant Frequency	Variant P-Value (approximate)	Amino Acid Change	CDS	product	Protein Effect	protein_id
G	31	31	1	A -> G	1638	SNP (transition)	78.80%	0		hypothetical protein CDS	hypothetical protein	None	KLI71769.1
A	34	34	1	G -> A	1654	SNP (transition)	78.90%	0		hypothetical protein CDS	hypothetical protein	None	KLI71769.1
C	61	61	1	G -> C	1722	SNP (transversion)	56.20%	0		hypothetical protein CDS	hypothetical protein	None	KLI71769.1
A	66	66	1	G -> A	1675	SNP (transition)	63.50%	0		hypothetical protein CDS	hypothetical protein	None	KLI71769.1
C	67	67	1	T -> C	1668	SNP (transition)	56.70%	0		hypothetical protein CDS	hypothetical protein	None	KLI71769.1
G	73	73	1	A -> G	1662	SNP (transition)	56.90%	0		hypothetical protein CDS	hypothetical protein	None	KLI71769.1
C	120	120	1	T -> C	540	SNP (transition)	52.00%	0	K -> E	hypothetical protein CDS	hypothetical protein	Substitution	KLI71769.1
A	7	7	1	G -> A	1807	SNP (transition)	40.20%	0		hypothetical protein CDS	hypothetical protein	None	KLI71769.1
AATC	10	13	3	CACG -> AATC	1727 -> 1796	Substitution	35.0% -> 35.7%	0	TV -> TI	hypothetical protein CDS	hypothetical protein	Substitution	KLI71769.1
G	22	22	1	A -> G	1688	SNP (transition)	38.70%	0		hypothetical protein CDS	hypothetical protein	None	KLI71769.1
G	31	31	1	A -> G	1638	SNP (transition)	78.80%	0		hypothetical protein CDS	hypothetical protein	None	KLI71769.1
A	34	34	1	G -> A	1654	SNP (transition)	78.90%	0		hypothetical protein CDS	hypothetical protein	None	KLI71769.1
T	52	52	1	C -> T	1677	SNP (transition)	34.80%	0		hypothetical protein CDS	hypothetical protein	None	KLI71769.1
C	55	55	1	T -> C	1699	SNP (transition)	33.30%	0		hypothetical protein CDS	hypothetical protein	None	KLI71769.1
C	61	61	1	G -> C	1722	SNP (transversion)	56.20%	0		hypothetical protein CDS	hypothetical protein	None	KLI71769.1
A	66	66	1	G -> A	1675	SNP (transition)	63.50%	0		hypothetical protein CDS	hypothetical protein	None	KLI71769.1
C	67	67	1	T -> C	1668	SNP (transition)	56.70%	0		hypothetical protein CDS	hypothetical protein	None	KLI71769.1
G	73	73	1	A -> G	1662	SNP (transition)	56.90%	0		hypothetical protein CDS	hypothetical protein	None	KLI71769.1

C	120	120	1	T -> C	540	SNP (transition)	52.00%	0	K -> E	hypothetical protein CDS	hypothetical protein	Substitution	KLI71769.1
C	175	175	1	T -> C	389	SNP (transition)	44.20%	0		hypothetical protein CDS	hypothetical protein	None	KLI71769.1

Contig 15

Name	Minimum	Maximum	Length	Change	Coverage	Polymorphism Type	Variant Frequency
C	1	1	1	A -> C	169	SNP (transversion)	96.40%
ATATCAG	4	10	7	CATATCA -> ATATCAG	169 -> 182	Substitution	87.4% -> 94.1%
ACG	12	14	3	GAC -> ACG	183	Substitution	87.40%
TCAG	17	20	4	GTCA -> TCAG	186 -> 190	Substitution	83.7% -> 85.5%
TA	22	23	2	GT -> TA	193	Substitution	81.3% -> 82.4%
CATCACTGCTCGCTCTGTCTAT	26	47	22	ACATCACTGCTCGCTCTGTCTA -> CATCACTGCTCGCTCTGTCTAT	191 -> 206	Substitution	70.4% -> 80.3%
CT	49	50	2	TC -> CT	201 -> 203	Substitution	69.7% -> 70.9%
GTCTCATA	52	59	8	TGTCTCAT -> GTCTCATA	191 -> 198	Substitution	63.6% -> 68.2%
C	62	62	1	A -> C	197	SNP (transversion)	62.90%
GCTGA	64	68	5	CGCTG -> GCTGA	199 -> 203	Substitution	59.1% -> 60.8%
CACAT	70	74	5	ACACA -> CACAT	198 -> 201	Substitution	55.3% -> 58.7%
GA	76	77	2	TG -> GA	205	Substitution	53.20%
G	80	80	1	A -> G	205	SNP (transition)	53.20%
C	82	82	1	G -> C	204	SNP (transversion)	52.00%
ATATA	84	88	5	CATAT -> ATATA	198 -> 205	Substitution	48.8% -> 50.5%
CG	92	93	2	AC -> CG	205	Substitution	47.30%
CTCAGTAC	95	102	8	GCTCAGTA -> CTCAGTAC	213 -> 216	Substitution	43.5% -> 45.1%
A	104	104	1	C -> A	216	SNP (transversion)	42.60%
CGT	107	109	3	ACG -> CGT	211 -> 216	Substitution	39.3% -> 41.2%
GCACA	111	115	5	TGCAC -> GCACA	213 -> 214	Substitution	37.9% -> 38.0%

G	117	117	1	A -> G	216	SNP (transition)	38.00%
T	119	119	1	G -> T	221	SNP (transversion)	37.10%
CTG	121	123	3	TCT -> CTG	221 -> 222	Substitution	36.2% -> 36.5%
TG	125	126	2	GT -> TG	222	Substitution	35.6% -> 36.0%
CTGA	128	131	4	GCTG -> CTGA	225 -> 228	Substitution	33.9% -> 35.1%
TCTCA	133	137	5	ATCTC -> TCTCA	226 -> 233	Substitution	31.8% -> 33.3%
T	139	139	1	A -> T	233	SNP (transversion)	31.80%
ACAC	141	144	4	TACA -> ACAC	228 -> 232	Substitution	30.3% -> 31.2%
ATCGCACT	146	153	8	CATCGCAC -> ATCGCACT	221 -> 226	Substitution	24.9% -> 27.6%

Contig 17

Name	Minimum	Maximum	Length	Change	Coverage	Polymorphism Type	Variant Frequency
G	22	22	1	A -> G	153	SNP (transition)	37.30%
A	36	36	1	G -> A	159	SNP (transition)	37.10%
C	72	73	2	AT -> C	179 -> 183	Deletion	40.4% -> 41.3%
A	216849	216848	0		84	Insertion	91.70%
T	216850	216850	1	A -> T	84	SNP (transversion)	96.40%
	216856	216857	2		346 -> 366	Deletion	51.6% -> 54.6%
A	216861	216861	1	T -> A	345	SNP (transversion)	35.10%
C	216862	216862	1	T -> C	351	SNP (transition)	53.80%
T	216864	216864	1	G -> T	353	SNP (transversion)	62.90%
T	216866	216866	1	G -> T	349	SNP (transversion)	55.00%
T	216868	216868	1	C -> T	349	SNP (transition)	53.90%
G	216871	216871	1	T -> G	343	SNP (transversion)	61.80%
A	216872	216872	1	T -> A	338	SNP (transversion)	54.10%
AA	216875	216875	1	C -> AA	333 -> 339	Insertion	49.6% -> 49.8%
T	216878	216878	1	C -> T	332	SNP (transition)	50.90%
G	216882	216882	1	T -> G	317	SNP (transversion)	53.30%

C	216946	216946	1	A -> C	214	SNP (transversion)	36.00%
	216955	216955	1		194	Deletion	25.80%
A	216958	216958	1	T -> A	186	SNP (transversion)	26.90%
G	217109	217109	1	A -> G	130	SNP (transition)	64.60%

Contig 19

Name	Minimum	Maximum	Length	Change	Coverage	Polymorphism Type	Variant Frequency
TTGTT	77	81	5	AAAAG -> TTGTT	110 -> 112	Substitution	24.5% -> 26.1%
AT	83	84	2	CC -> AT	112 -> 115	Substitution	26.8% -> 28.7%
AATCAA	86	91	6	TCGGGC -> AATCAA	126 -> 128	Substitution	34.9% -> 35.9%
GT	93	94	2	AG -> GT	128 -> 132	Substitution	34.8% -> 35.9%
GC	96	97	2	CA -> GC	135 -> 136	Substitution	34.6% -> 34.8%
A	99	99	1	T -> A	139	SNP (transversion)	34.50%
T	197	197	1	C -> T	148	SNP (transition)	26.40%

Contig 22

Name	Minimum	Maximum	Length	Change	Coverage	Polymorphism Type	Variant Frequency	Variant P-Value (approximate)	Amino Acid Change	CDS	product	Protein Effect	protein_id
T	31636	31636	1	C -> T	163	SNP (transition)	32.50%	4.60E-148		hypothetical protein CDS	hypothetical protein	None	KLI70765.1

Contig 23

Name	Minimum	Maximum	Length	Change	Coverage	Polymorphism Type	Variant Frequency
CT	2	3	2	TA -> CT	252 -> 253	Substitution	38.1% -> 38.3%

T	6	6	1	G -> T	257	SNP (transversion)	48.60%
T	68	68	1	G -> T	130	SNP (transversion)	31.50%
C	70	70	1	T -> C	130	SNP (transition)	31.50%
GTT	72	74	3	TGC -> GTT	130	Substitution	31.50%
C	78	78	1	A -> C	135	SNP (transversion)	40.70%
T	79	79	1	A -> T	135	SNP (transversion)	30.40%
T	80	80	1	G -> T	135	SNP (transversion)	40.00%
GC	82	83	2	TT -> GC	135	Substitution	29.60%
CAAAA	85	89	5	TCTCT -> CAAAA	135	Substitution	30.40%
GGA	92	94	3	AAG -> GGA	136 -> 138	Substitution	29.0% -> 29.4%
C	98	102	5	AGGTG -> C	142 -> 144	Deletion	28.5% -> 28.9%
T	107	107	1	C -> T	148	SNP (transition)	36.50%
G	114	114	1	T -> G	142	SNP (transversion)	26.10%
A	118	118	1	G -> A	148	SNP (transition)	25.00%
A	119	119	1	T -> A	148	SNP (transversion)	33.80%
G	120	120	1	T -> G	148	SNP (transversion)	25.00%
T	121	121	1	G -> T	148	SNP (transversion)	33.10%

Contig 24

Name	Minimum	Maximum	Length	Change	Coverage	Polymorphism Type	Variant Frequency
	195668	195668	1	(A)8 -> (A)7	22	Deletion (tandem repeat)	95.50%
T	74	74	1	C -> T	596	SNP (transition)	31.40%
T	163	163	1	A -> T	451	SNP (transversion)	34.10%
T	164	164	1	A -> T	446	SNP (transversion)	39.20%
T	168	168	1	A -> T	410	SNP (transversion)	33.70%
G	169	169	1	A -> G	409	SNP (transition)	47.70%
C	170	170	1	G -> C	404	SNP (transversion)	31.90%
T	170	170	1	G -> T	404	SNP (transversion)	29.50%
G	171	171	1	A -> G	393	SNP (transition)	26.70%
T	171	171	1	A -> T	393	SNP (transversion)	60.60%

C	172	172	1	A -> C	381	SNP (transversion)	27.80%
T	172	172	1	A -> T	381	SNP (transversion)	52.20%
T	174	174	1	C -> T	365	SNP (transition)	38.10%
T	175	175	1	C -> T	363	SNP (transition)	42.70%
T	176	176	1	C -> T	360	SNP (transition)	66.40%
A	178	178	1	C -> A	352	SNP (transversion)	31.50%
T	178	178	1	C -> T	352	SNP (transition)	27.60%
T	180	180	1	C -> T	342	SNP (transition)	64.60%
A	181	181	1	G -> A	332	SNP (transition)	50.30%
T	182	182	1	A -> T	325	SNP (transversion)	57.20%
C	183	183	1	A -> C	320	SNP (transversion)	35.60%
A	185	185	1	G -> A	303	SNP (transition)	63.70%
T	188	188	1	C -> T	252	SNP (transition)	55.60%
A	190	190	1	G -> A	245	SNP (transition)	47.30%
A	191	191	1	G -> A	237	SNP (transition)	31.20%
T	192	192	1	C -> T	232	SNP (transition)	31.90%
A	193	193	1	T -> A	225	SNP (transversion)	42.70%
G	193	193	1	T -> G	225	SNP (transversion)	31.60%
A	194	194	1	T -> A	213	SNP (transversion)	38.00%
C	194	194	1	T -> C	213	SNP (transition)	26.30%
A	195	195	1	T -> A	207	SNP (transversion)	44.00%
A	196	196	1	T -> A	201	SNP (transversion)	53.70%
A	197	197	1	T -> A	193	SNP (transversion)	42.50%
C	197	197	1	T -> C	193	SNP (transition)	36.30%
A	198	198	1	T -> A	189	SNP (transversion)	36.00%
C	198	198	1	T -> C	189	SNP (transition)	28.00%
A	199	199	1	G -> A	184	SNP (transition)	35.90%
G	201	201	1	A -> G	177	SNP (transition)	32.20%
T	201	201	1	A -> T	177	SNP (transversion)	36.70%
G	202	202	1	T -> G	175	SNP (transversion)	52.60%
C	204	204	1	T -> C	168	SNP (transition)	25.60%

G	204	204	1	T -> G	168	SNP (transversion)	25.60%
A	206	206	1	T -> A	156	SNP (transversion)	38.50%
TG	208	209	2	GA -> TG	129 -> 139	Substitution	38.8% -> 40.3%
G	213	213	1	T -> G	116	SNP (transversion)	28.40%
T	215	215	1	A -> T	111	SNP (transversion)	40.50%
G	221	221	1	A -> G	87	SNP (transition)	33.30%
A	227	227	1	C -> A	81	SNP (transversion)	28.40%
	195668	195668	1	(A)8 -> (A)7	22	Deletion (tandem repeat)	95.50%

Contig 27

Name	Minimum	Maximum	Length	Change	Coverage	Polymorphism Type	Variant Frequency	Variant P-Value (approximate)	CDS	product	Protein Effect	protein_id	Amino Acid Change
ACAGCAACAGCAACAGCAACAGCAACAGCAACAGCAACAGCA	27343	27342	0	(AACAGC)13 -> (AACAGC)20	56 -> 104	Insertion (tandem repeat)	94.8% -> 97.1%	4.00E-163	hypothetical protein CDS	hypothetical protein	Insertion	KLI70436.1	Q -> QQQQQQQQQQQQQQ
ACAGCAACAGCAACAGCAACAGCAACAGCAACAGCAACAGCA	27343	27342	0	(AACAGC)13 -> (AACAGC)20	56 -> 104	Insertion (tandem repeat)	94.8% -> 97.1%	4.00E-163	hypothetical protein CDS	hypothetical protein	Insertion	KLI70436.1	Q -> QQQQQQQQQQQQQQ

Contig 28

Name	Minimum	Maximum	Length	Change	Coverage	Polymorphism Type	Variant Frequency	Variant P-Value (approximate)	CDS	product	Protein Effect	protein_id	Amino Acid Change

C	10129	10129	1	G -> C	460	SNP (transversion)	65.00%	0	calcium-binding protein CDS	calcium-binding protein	None	KLI70416.1
T	10159	10159	1	C -> T	469	SNP (transition)	51.80%	0	calcium-binding protein CDS	calcium-binding protein	None	KLI70416.1
G	10405	10405	1	C -> G	535	SNP (transversion)	57.40%	0	calcium-binding protein CDS	calcium-binding protein	None	KLI70416.1
G	10441	10441	1	A -> G	566	SNP (transition)	67.50%	0	calcium-binding protein CDS	calcium-binding protein	None	KLI70416.1
C	10498	10498	1	T -> C	534	SNP (transition)	73.00%	0	calcium-binding protein CDS	calcium-binding protein	None	KLI70416.1
T	10532	10532	1	C -> T	110	SNP (transition)	58.20%	5.60E-187	calcium-binding protein CDS	calcium-binding protein	None	KLI70416.1
G	10120	10120	1	T -> G	476	SNP (transversion)	38.40%	0	calcium-binding protein CDS	calcium-binding protein	None	KLI70416.1
C	10129	10129	1	G -> C	460	SNP (transversion)	65.00%	0	calcium-binding protein CDS	calcium-binding protein	None	KLI70416.1
T	10145	10145	1	C -> T	452	SNP (transition)	48.00%	0	calcium-binding protein CDS	calcium-binding protein	None	KLI70416.1
C	10153	10153	1	T -> C	454	SNP (transition)	48.90%	0	calcium-binding protein CDS	calcium-binding protein	None	KLI70416.1
T	10159	10159	1	C -> T	469	SNP (transition)	51.80%	0	calcium-binding protein CDS	calcium-binding protein	None	KLI70416.1
A	10204	10204	1	C -> A	433	SNP (transversion)	37.60%	0	calcium-binding protein CDS	calcium-binding protein	None	KLI70416.1
C	10273	10273	1	T -> C	511	SNP (transition)	41.10%	0	calcium-binding protein CDS	calcium-binding protein	None	KLI70416.1
G	10282	10282	1	C -> G	494	SNP (transversion)	38.10%	0	calcium-binding protein CDS	calcium-binding protein	None	KLI70416.1
T	10351	10351	1	A -> T	497	SNP (transversion)	30.80%	0	calcium-binding protein CDS	calcium-binding protein	None	KLI70416.1
T	10360	10360	1	C -> T	487	SNP (transition)	27.50%	0	calcium-binding protein CDS	calcium-binding protein	None	KLI70416.1
A	10369	10369	1	G -> A	485	SNP (transition)	41.90%	0	calcium-binding protein CDS	calcium-binding protein	None	KLI70416.1
A	10387	10387	1	G -> A	498	SNP (transition)	39.40%	0	calcium-binding protein CDS	calcium-binding protein	None	KLI70416.1
C	10396	10396	1	T -> C	548	SNP (transition)	48.40%	0	calcium-binding protein CDS	calcium-binding protein	None	KLI70416.1
G	10405	10405	1	C -> G	535	SNP (transversion)	57.40%	0	calcium-binding protein CDS	calcium-binding protein	None	KLI70416.1

G	10414	10414	1	A -> G	523	SNP (transition)	34.00%	0	calcium-binding protein CDS	calcium-binding protein	None	KLI70416.1	
T	10429	10429	1	C -> T	557	SNP (transition)	42.50%	0	calcium-binding protein CDS	calcium-binding protein	None	KLI70416.1	
T	10432	10432	1	C -> T	565	SNP (transition)	27.60%	0	calcium-binding protein CDS	calcium-binding protein	None	KLI70416.1	
GT	10434	10435	2	CG -> GT	562	Substitution	29.0% -> 29.2%	0	calcium-binding protein CDS	calcium-binding protein	Substitution	KLI70416.1	T -> S
G	10441	10441	1	A -> G	566	SNP (transition)	67.50%	0	calcium-binding protein CDS	calcium-binding protein	None	KLI70416.1	
C	10444	10444	1	A -> C	576	SNP (transversion)	45.00%	0	calcium-binding protein CDS	calcium-binding protein	None	KLI70416.1	
T	10444	10444	1	A -> T	576	SNP (transversion)	28.10%	0	calcium-binding protein CDS	calcium-binding protein	None	KLI70416.1	
C	10447	10447	1	T -> C	568	SNP (transition)	32.40%	0	calcium-binding protein CDS	calcium-binding protein	None	KLI70416.1	
ACC	10481	10483	2	TCA -> ACC	539 -> 557	Substitution	38.2% -> 38.4%	0	calcium-binding protein CDS	calcium-binding protein	Substitution	KLI70416.1	S -> T
C	10486	10486	1	T -> C	537	SNP (transition)	34.60%	0	calcium-binding protein CDS	calcium-binding protein	None	KLI70416.1	
C	10498	10498	1	T -> C	534	SNP (transition)	73.00%	0	calcium-binding protein CDS	calcium-binding protein	None	KLI70416.1	
G	10510	10510	1	C -> G	136	SNP (transversion)	43.40%	7.10E-174	calcium-binding protein CDS	calcium-binding protein	Substitution	KLI70416.1	D -> E
C	10516	10516	1	G -> C	119	SNP (transversion)	49.60%	1.50E-172	calcium-binding protein CDS	calcium-binding protein	None	KLI70416.1	
T	10532	10532	1	C -> T	110	SNP (transition)	58.20%	5.60E-187	calcium-binding protein CDS	calcium-binding protein	None	KLI70416.1	
A	10534	10534	1	G -> A	107	SNP (transition)	38.30%	2.60E-110	calcium-binding protein CDS	calcium-binding protein	None	KLI70416.1	
C	10540	10540	1	T -> C	102	SNP (transition)	35.30%	1.20E-102	calcium-binding protein CDS	calcium-binding protein	None	KLI70416.1	
T	10546	10546	1	C -> T	94	SNP (transition)	29.80%	1.00E-77	calcium-binding protein CDS	calcium-binding protein	None	KLI70416.1	
AG	10549	10550	2	GT -> AG	93 -> 94	Substitution	29.0% -> 29.8%	6.00E-72	calcium-binding protein CDS	calcium-binding protein	Substitution	KLI70416.1	ES -> EA

Contig 29

Name	Minimum	Maximum	Length	Change	Coverage	Polymorphism Type	Variant Frequency
G	133	133	1	A -> G	227	SNP (transition)	36.60%
T	135	135	1	A -> T	233	SNP (transversion)	35.60%
T	137	136	0		221	Insertion	27.60%
T	141	141	1	C -> T	207	SNP (transition)	30.00%
T	142	142	1	C -> T	206	SNP (transition)	40.30%
C	143	143	1	T -> C	206	SNP (transition)	53.90%
C	145	145	1	T -> C	207	SNP (transition)	52.70%
CG	147	148	2	TC -> CG	208 -> 210	Substitution	29.5% -> 29.8%
A	151	151	1	G -> A	206	SNP (transition)	39.30%
C	154	154	1	T -> C	206	SNP (transition)	41.70%
C	165	165	1	A -> C	186	SNP (transversion)	37.10%
T	168	168	1	G -> T	170	SNP (transversion)	53.50%
A	250	250	1	T -> A	147	SNP (transversion)	51.70%
AG	252	253	2	TT -> AG	146	Substitution	51.40%
TCTC	255	258	4	ATAA -> TCTC	144 -> 150	Substitution	48.0% -> 50.0%
C	260	260	1	T -> C	148	SNP (transition)	47.30%
GT	262	263	2	AA -> GT	144 -> 145	Substitution	46.5% -> 46.9%
C	265	265	1	G -> C	140	SNP (transversion)	48.60%
	266	279	14		137 -> 161	Deletion	33.5% -> 39.4%
T	281	281	1	C -> T	157	SNP (transition)	46.50%
GT	286	287	2	AA -> GT	169 -> 172	Substitution	44.8% -> 45.6%
C	290	290	1	T -> C	179	SNP (transition)	43.60%
AC	291	292	2	GA -> AC	181	Substitution	51.40%

AT	294	295	2	GC -> AT	194 -> 195	Substitution	37.9% -> 38.1%
	298	299	2		212	Deletion	35.80%

Contig 31

Name	Minimum	Maximum	Length	Change	Coverage	Polymorphism Type	Variant Frequency	Variant P-Value (approximate)	Amino Acid Change	CDS	product	Protein Effect	protein_id
	16050	16055	6	(TCTGCT)11 -> (TCTGCT)10	586 -> 587	Deletion (tandem repeat)	98.6% -> 98.8%	0	EAE -> E	cell division protein FtsY CDS	cell division protein FtsY	Deletion	KLI70360.1
TCTGCT	16116	16115	0	(TCTGCT)11 -> (TCTGCT)12	627 -> 637	Insertion (tandem repeat)	97.1% -> 97.2%	0	P -> PAE	cell division protein FtsY CDS	cell division protein FtsY	Insertion	KLI70360.1
	16050	16055	6	(TCTGCT)11 -> (TCTGCT)10	586 -> 587	Deletion (tandem repeat)	98.6% -> 98.8%	0	EAE -> E	cell division protein FtsY CDS	cell division protein FtsY	Deletion	KLI70360.1
TCTGCT	16116	16115	0	(TCTGCT)11 -> (TCTGCT)12	627 -> 637	Insertion (tandem repeat)	97.1% -> 97.2%	0	P -> PAE	cell division protein FtsY CDS	cell division protein FtsY	Insertion	KLI70360.1

Contig 34

Name	Minimum	Maximum	Length	Change	Coverage	Polymorphism Type	Variant Frequency	Variant P-Value (approximate)	Amino Acid Change	CDS	product	Protein Effect	protein_id
G	3957	3957	1	A -> G	165	SNP (transition)	48.50%	2.60E-240		nucleoside permease CDS	nucleoside permease	None	KLI70272.1
AT	3996	3997	2	GC -> AT	145 -> 146	Substitution	47.3% -> 47.6%	1.50E-199	RH -> RY	nucleoside permease CDS	nucleoside permease	Substitution	KLI70272.1
G	4024	4024	1	T -> G	116	SNP (transversion)	39.70%	5.00E-129	E -> D	nucleoside permease CDS	nucleoside permease	Substitution	KLI70272.1

Contig 38

Name	Minimum	Maximum	Length	Change	Coverage	Polymorphism Type	Variant Frequency	Variant P-Value (approximate)
G	22	22	1	A -> G	147	SNP (transition)	28.60%	1.10E-110
G	30	30	1	A -> G	144	SNP (transition)	25.70%	2.00E-99
A	45	45	1	T -> A	116	SNP (transversion)	25.00%	2.70E-66

Contig 40

Name	Minimum	Maximum	Length	Change	Coverage	Polymorphism Type	Variant Frequency	Variant P-Value (approximate)
C	66081	66081	1	G -> C	133	SNP (transversion)	26.30%	1.50E-87
G	66087	66087	1	A -> G	130	SNP (transition)	26.90%	5.90E-95
A	66092	66092	1	G -> A	128	SNP (transition)	26.60%	1.20E-88
T	66114	66114	1	C -> T	121	SNP (transition)	28.90%	3.20E-96

Contig 46

Name	Minimum	Maximum	Length	Change	Coverage	Polymorphism Type	Variant Frequency
A	1890	1890	1	G -> A	160	SNP (transition)	35.60%
G	1920	1920	1	A -> G	161	SNP (transition)	44.70%
A	1999	1999	1	C -> A	194	SNP (transversion)	29.40%

Contig 50

Name	Minimum	Maximum	Length	Change	Coverage	Polymorphism Type	Variant Frequency
A	63513	63513	1	T -> A	174	SNP (transversion)	25.30%

Contig 51

Name	Minimum	Maximum	Length	Change	Coverage	Polymorphism Type	Variant Frequency
AT	2815	2816	2	CG -> AT	132	Substitution	61.40%
A	2820	2820	1	G -> A	133	SNP (transition)	60.90%
T	2846	2846	1	C -> T	139	SNP (transition)	43.90%
T	2860	2860	1	C -> T	144	SNP (transition)	36.80%

Contig 53

Name	Minimum	Maximum	Length	Change	Coverage	Polymorphism Type	Variant Frequency
A	36	36	1	G -> A	284	SNP (transition)	33.50%
T	856	856	1	A -> T	3113	SNP (transversion)	26.10%
C	1196	1196	1	A -> C	2706	SNP (transversion)	48.90%
A	2748	2748	1	C -> A	3893	SNP (transversion)	32.80%
A	2757	2757	1	G -> A	3992	SNP (transition)	32.80%
C	2759	2759	1	T -> C	3921	SNP (transition)	32.90%

Contig 54

Name	Minimum	Maximum	Length	Change	Coverage	Polymorphism Type	Variant Frequency
A	46	46	1	G -> A	650	SNP (transition)	26.80%
G	61	61	1	A -> G	611	SNP (transition)	35.50%
T	72	72	1	C -> T	550	SNP (transition)	36.90%
G	1153	1153	1	A -> G	4320	SNP (transition)	30.70%
C	1173	1173	1	T -> C	4389	SNP (transition)	37.30%
G	1550	1550	1	A -> G	2697	SNP (transition)	26.40%
A	1679	1678	0	(A)2 -> (A)3	822	Insertion (tandem repeat)	31.50%
CGAA	1707	1710	4	TTTC -> CGAA	502 -> 510	Substitution	29.8% -> 30.1%

Contig 57

Name	Minimum	Maximum	Length	Change	Coverage	Polymorphism Type	Variant Frequency	Variant P-Value (approximate)	Amino Acid Change	CDS	product	Protein Effect	protein_id
G	159	159	1	T -> G	275	SNP (transversion)	53.50%	0		RTX toxin CDS	RTX toxin	None	KLI69968.1
G	173	173	1	A -> G	307	SNP (transition)	51.50%	0	D -> G	RTX toxin CDS	RTX toxin	Substitution	KLI69968.1
A	201	201	1	G -> A	344	SNP (transition)	54.70%	0		RTX toxin CDS	RTX toxin	None	KLI69968.1
C	1122	1122	1	A -> C	404	SNP (transversion)	53.50%	0		RTX toxin CDS	RTX toxin	None	KLI69968.1
A	1159	1159	1	G -> A	417	SNP (transition)	51.60%	0	A -> T	RTX toxin CDS	RTX toxin	Substitution	KLI69968.1

G	1191	1191	1	A -> G	404	SNP (transition)	50.50%	0		RTX toxin CDS	RTX toxin	None	KLI69968.1
T	1323	1323	1	C -> T	391	SNP (transition)	60.90%	0		RTX toxin CDS	RTX toxin	None	KLI69968.1
C	1353	1353	1	T -> C	397	SNP (transition)	60.50%	0		RTX toxin CDS	RTX toxin	None	KLI69968.1
G	159	159	1	T -> G	275	SNP (transversion)	53.50%	0		RTX toxin CDS	RTX toxin	None	KLI69968.1
G	173	173	1	A -> G	307	SNP (transition)	51.50%	0	D -> G	RTX toxin CDS	RTX toxin	Substitution	KLI69968.1
A	201	201	1	G -> A	344	SNP (transition)	54.70%	0		RTX toxin CDS	RTX toxin	None	KLI69968.1
G	363	363	1	A -> G	379	SNP (transition)	47.80%	0		RTX toxin CDS	RTX toxin	None	KLI69968.1
G	427	427	1	A -> G	380	SNP (transition)	49.70%	0	I -> V	RTX toxin CDS	RTX toxin	Substitution	KLI69968.1
C	437	437	1	T -> C	366	SNP (transition)	49.70%	0	L -> S	RTX toxin CDS	RTX toxin	Substitution	KLI69968.1
G	723	723	1	A -> G	351	SNP (transition)	49.60%	0		RTX toxin CDS	RTX toxin	None	KLI69968.1
G	728	728	1	T -> G	342	SNP (transversion)	49.40%	0	V -> G	RTX toxin CDS	RTX toxin	Substitution	KLI69968.1
A	745	745	1	G -> A	334	SNP (transition)	49.10%	0	A -> T	RTX toxin CDS	RTX toxin	Substitution	KLI69968.1
A	840	840	1	G -> A	306	SNP (transition)	48.40%	0		RTX toxin CDS	RTX toxin	None	KLI69968.1
C	986	986	1	T -> C	284	SNP (transition)	47.90%	0	I -> T	RTX toxin CDS	RTX toxin	Substitution	KLI69968.1
T	1019	1019	1	C -> T	311	SNP (transition)	45.70%	0	T -> M	RTX toxin CDS	RTX toxin	Substitution	KLI69968.1
C	1122	1122	1	A -> C	404	SNP (transversion)	53.50%	0		RTX toxin CDS	RTX toxin	None	KLI69968.1
A	1159	1159	1	G -> A	417	SNP (transition)	51.60%	0	A -> T	RTX toxin CDS	RTX toxin	Substitution	KLI69968.1
G	1191	1191	1	A -> G	404	SNP (transition)	50.50%	0		RTX toxin CDS	RTX toxin	None	KLI69968.1
T	1323	1323	1	C -> T	391	SNP (transition)	60.90%	0		RTX toxin CDS	RTX toxin	None	KLI69968.1
C	1353	1353	1	T -> C	397	SNP (transition)	60.50%	0		RTX toxin CDS	RTX toxin	None	KLI69968.1
T	1418	1418	1	C -> T	371	SNP (transition)	39.90%	0	S -> L	RTX toxin CDS	RTX toxin	Substitution	KLI69968.1
G	1425	1425	1	A -> G	374	SNP (transition)	40.90%	0		RTX toxin CDS	RTX toxin	None	KLI69968.1

Contig 58

Name	Minimum	Maximum	Length	Change	Coverage	Polymorphism Type	Variant Frequency
TTAA	1	4	4	AACC -> TTAA	2	Substitution	100.00%
TA	6	7	2	AT -> TA	2	Substitution	100.00%
AAC	9	11	3	TCA -> AAC	2	Substitution	100.00%
CT	13	14	2	GA -> CT	2	Substitution	100.00%

Contig 59

Name	Minimum	Maximum	Length	Change	Coverage	Polymorphism Type	Variant Frequency
ACAAA	17	21	5	GTTGT -> ACAAA	75 -> 76	Substitution	54.7% -> 55.3%
T	22	22	1	G -> T	66	SNP (transversion)	69.70%
T	23	23	1	G -> T	65	SNP (transversion)	55.40%
TCA	25	27	3	CGG -> TCA	59 -> 63	Substitution	54.1% -> 57.4%
C	28	28	1	T -> C	60	SNP (transition)	65.00%
TA	29	30	2	AT -> TA	58 -> 60	Substitution	53.3% -> 53.4%
AC	33	34	2	TA -> AC	52 -> 53	Substitution	53.8% -> 54.7%
T	35	35	1	A -> T	51	SNP (transversion)	66.70%
T	36	36	1	G -> T	50	SNP (transversion)	54.00%
AG	37	38	2	GA -> AG	46 -> 49	Substitution	67.3% -> 69.6%
T	39	39	1	G -> T	47	SNP (transversion)	55.30%
T	41	41	1	A -> T	47	SNP (transversion)	66.00%
T	44	44	1	G -> T	46	SNP (transversion)	54.30%
A	45	45	1	T -> A	45	SNP (transversion)	64.40%
T	47	47	1	C -> T	33	SNP (transition)	39.40%
AT	48	49	2	GA -> AT	30	Substitution	50.00%
TG	50	51	2	CT -> TG	29	Substitution	31.00%

Contig 60

Name	Minimum	Maximum	Length	Change	Coverage	Polymorphism Type	Variant Frequency
C	98	98	1	T -> C	116	SNP (transition)	43.10%

Contig 61

Name	Minimum	Maximum	Length	Change	Coverage	Polymorphism Type	Variant Frequency
A	8	8	1	T -> A	3	SNP (transversion)	100.00%
TC	11	12	2	CA -> TC	3	Substitution	66.70%
C	29	29	1	T -> C	3	SNP (transition)	66.70%

Contig 62

Name	Minimum	Maximum	Length	Change	Coverage	Polymorphism Type	Variant Frequency
CG	2723	2724	2	TT -> CG	30	Substitution	100.00%
T	2728	2728	1	C -> T	30	SNP (transition)	100.00%
T	2744	2744	1	A -> T	23	SNP (transversion)	100.00%
A	2747	2747	1	G -> A	23	SNP (transition)	100.00%

Contig 63

Name	Minimum	Maximum	Length	Change	Coverage	Polymorphism Type	Variant Frequency	Variant P-Value (approximate)	Amino Acid Change	CDS	product	Protein Effect	protein_id
GCAACA GCAACA	89	88	0	(AGCAAC)14 -> (AGCAAC)16	144 -> 161	Insertion (tandem repeat)	99.3% -> 99.4%	0	Q -> QQQQ Q	hypothetical protein CDS	hypothetical protein	Insertion	KLI69 899.1
GCAACA GCAACA	89	88	0	(AGCAAC)14 -> (AGCAAC)16	144 -> 161	Insertion (tandem repeat)	99.3% -> 99.4%	0	Q -> QQQQ Q	hypothetical protein CDS	hypothetical protein	Insertion	KLI69 899.1
