# Supplementary material for: Perturbation of Quorum Sensing after the Acquisition of Bacteriophage Resistance Could Contribute to Novel Traits in Vibrio alginolyticus
Source: Microorganisms. 2023 Sep 10;11(9):2273. doi: 10.3390/microorganisms11092273 (PMC10535087; doi:10.3390/microorganisms11092273)
Supplement: Supplementary file 1 [file microorganisms-11-02273-s001.zip › Table S4.SNPs corresponding to the bacteriophage resistant strain VaAthena1_B.pdf]

Skliros et al., 2023. Supplemental Table S4. Single Nucleotide Polymorphisms of resistant strain **VaAthena1_B** for each Contig separately. Tables show Nucleotide shift, position, change, polymorphism, type, variant frequency and in the case of a mutation in a CDS product, protein, effect and protein_id.

Contig 3

Name	Minimum	Maximum	Length	Change	Coverage	Polymorphism Type	Variant Frequency
A	2716	2716	1	T -> A	5	SNP (transversion)	40.00%

Contig 6

Name	Minimum	Maximum	Length	Change	Coverage	Polymorphism Type	Variant Frequency
AT	49	50	2	TC -> AT	155 -> 156	Substitution	27.1% -> 27.6%
T	52	52	1	C -> T	160	SNP (transition)	28.70%
T	64	64	1	A -> T	183	SNP (tran	32.20%

						sversion)	
T	152	151	0		236	Insertion	39.40%
G	155	155	1	A -> G	235	SNP (transition)	39.10%
TCA	157	159	3	ATT -> TCA	230 -> 238	Substitution	38.7% -> 39.6%
GT	162	163	2	TC -> GT	226 -> 229	Substitution	40.2% -> 40.7%
TTAAAATGGCTACGTAGCTCAGCTGGTTAGAGCACATCACTCATAATGATGGGGTCACAGGTTCGAATCCCGTCGTAGCCACCAT	165	165	1	A -> TTAAAATGGCTACGTAGCTCAGCTGGTTAGAGCACATCACTCATAATGATGGGGTCACAGGTTCGAATCCCGTCGTAGCCACCAT	195 -> 223	Insertion	30.8% -> 38.3%
A	166	165	0		189	Insertion	31.70%
TT	166	165	0		181 -> 182	Insertion	28.7% -> 29.1%
T	166	165	0		179	Insertion	27.90%

T	166	165	0		172	Insertion	25.00%
AA	310	311	2	CC -> AA	142 -> 166	Substitution	30.3% -> 38.0%
T	390	390	1	C -> T	186	SNP (transition)	54.80%
C	397	397	1	T -> C	158	SNP (transition)	36.70%
C	420	420	1	T -> C	351	SNP (transition)	51.00%
A	428	428	1	T -> A	391	SNP (transversion)	46.50%
A	436	436	1	T -> A	436	SNP (transversion)	48.60%
C	437	437	1	T -> C	436	SNP (transition)	31.40%

Contig 7

Name	Minimum	Maximum	Length	Change	Coverage	Polymorphism Type	Variant Frequency	Variant P-Value (approximate)	Amino Acid Change	CDS	product	Protein Effect	protein_id
C	216879	216879	1	T -> C	418	SNP (transition)	41.40 %	0		calcium-binding protein CDS	calcium-binding protein	None	KLI72868.1
C	216885	216885	1	A -> C	421	SNP (transversion)	40.60 %	0		calcium-binding protein CDS	calcium-binding protein	None	KLI72868.1
C	216888	216888	1	G -> C	425	SNP (transversion)	41.20 %	0		calcium-binding protein CDS	calcium-binding protein	None	KLI72868.1
T	216921	216921	1	C -> T	401	SNP (transition)	39.70 %	0		calcium-binding protein CDS	calcium-binding protein	None	KLI72868.1
T	216924	216924	1	C -> T	399	SNP (transition)	39.30 %	0		calcium-binding protein CDS	calcium-binding protein	None	KLI72868.1
C	216927	216927	1	G -> C	401	SNP (transversion)	38.90 %	0		calcium-binding protein CDS	calcium-binding protein	None	KLI72868.1
A	216942	216942	1	T -> A	385	SNP (transversion)	33.80 %	0		calcium-binding protein CDS	calcium-binding protein	None	KLI72868.1
G	216951	216951	1	A -> G	414	SNP (transition)	29.50 %	4.0E-320		calcium-binding protein CDS	calcium-binding protein	None	KLI72868.1

Contig 13

Name	Minimum	Maximum	Length	Change	Coverage	Polymorphism Type	Variant Frequency

A	286321	286321	1	G -> A	483	SNP (transition)	32.10%

Contig 14

Name	Minimum	Maximum	Length	Change	Coverage	Polymorphism Type	Variant Frequency	Variant P-Value (approximate)	Amino Acid Change	CDS	product	Protein Effect	protein_id
A	7	7	1	G -> A	3598	SNP (transition)	38.20%	0		hypothetical protein CDS	hypothetical protein	None	KLI71769.1
AATC	10	13	3	CACG -> AATC	3499 -> 3602	Substitution	35.5% -> 35.9%	0	TV -> TI	hypothetical protein CDS	hypothetical protein	Substitution	KLI71769.1
G	22	22	1	A -> G	3473	SNP (transition)	41.70%	0		hypothetical protein CDS	hypothetical protein	None	KLI71769.1
G	31	31	1	A -> G	3465	SNP (transition)	81.70%	0		hypothetical protein CDS	hypothetical protein	None	KLI71769.1
A	34	34	1	G -> A	3491	SNP (transition)	81.90%	0		hypothetical protein CDS	hypothetical protein	None	KLI71769.1
T	52	52	1	C -> T	3543	SNP (transition)	37.80%	0		hypothetical protein CDS	hypothetical protein	None	KLI71769.1
C	55	55	1	T -> C	3588	SNP (transition)	37.30%	0		hypothetical protein CDS	hypothetical protein	None	KLI71769.1
C	61	61	1	G -> C	3620	SNP (transversion)	63.30%	0		hypothetical protein CDS	hypothetical protein	None	KLI71769.1

AC	66	67	2	GT -> AC	3550 -> 3571	Substitution	63.4% -> 63.6%	0		hypothetical protein CDS	hypothetical protein	None	KLI71769.1
G	73	73	1	A -> G	3569	SNP (transition)	63.50%	0		hypothetical protein CDS	hypothetical protein	None	KLI71769.1
C	120	120	1	T -> C	805	SNP (transition)	54.30%	0	K -> E	hypothetical protein CDS	hypothetical protein	Substitution	KLI71769.1
C	175	175	1	T -> C	578	SNP (transition)	49.70%	0		hypothetical protein CDS	hypothetical protein	None	KLI71769.1

Contig 15

Name	Minimum	Maximum	Length	Change	Coverage	Polymorphism Type	Variant Frequency
C	1	1	1	A -> C	169	SNP (transversion)	96.40%
ATATCAG	4	10	7	CATATCA -> ATATCAG	169 -> 182	Substitution	87.4% -> 94.1%
ACG	12	14	3	GAC -> ACG	183	Substitution	87.40%
TCAG	17	20	4	GTCA -> TCAG	186 -> 190	Substitution	83.7% -> 85.5%
TA	22	23	2	GT -> TA	193	Substitution	81.3% -> 82.4%
CATCACTGCTCGCTCTGTCTAT	26	47	22	ACATCACTGCTCGCTCTGTCTA -> CATCACTGCTCGCTCTGTCTAT	191 -> 206	Substitution	70.4% -> 80.3%
CT	49	50	2	TC -> CT	201 -> 203	Substitution	69.7% -> 70.9%

GTCTCATA	52	59	8	TGTCTCAT -> GTCTCATA	191 -> 198	Substitution	63.6% -> 68.2%
C	62	62	1	A -> C	197	SNP (transversion)	62.90%
GCTGA	64	68	5	CGCTG -> GCTGA	199 -> 203	Substitution	59.1% -> 60.8%
CACAT	70	74	5	ACACA -> CACAT	198 -> 201	Substitution	55.3% -> 58.7%
GA	76	77	2	TG -> GA	205	Substitution	53.20%
G	80	80	1	A -> G	205	SNP (transition)	53.20%
C	82	82	1	G -> C	204	SNP (transversion)	52.00%
ATATA	84	88	5	CATAT -> ATATA	198 -> 205	Substitution	48.8% -> 50.5%
CG	92	93	2	AC -> CG	205	Substitution	47.30%
CTCAGTAC	95	102	8	GCTCAGTA -> CTCAGTAC	213 -> 216	Substitution	43.5% -> 45.1%
A	104	104	1	C -> A	216	SNP (transversion)	42.60%
CGT	107	109	3	ACG -> CGT	211 -> 216	Substitution	39.3% -> 41.2%
GCACA	111	115	5	TGCAC -> GCACA	213 -> 214	Substitution	37.9% -> 38.0%
G	117	117	1	A -> G	216	SNP (transition)	38.00%
T	119	119	1	G -> T	221	SNP (transversion)	37.10%
CTG	121	123	3	TCT -> CTG	221 -> 222	Substitution	36.2% -> 36.5%
TG	125	126	2	GT -> TG	222	Substitution	35.6% -> 36.0%

CTGA	128	131	4	GCTG -> CTGA	225 -> 228	Substitution	33.9% -> 35.1%
TCTCA	133	137	5	ATCTC -> TCTCA	226 -> 233	Substitution	31.8% -> 33.3%
T	139	139	1	A -> T	233	SNP (transversion)	31.80%
ACAC	141	144	4	TACA -> ACAC	228 -> 232	Substitution	30.3% -> 31.2%
ATCGCACT	146	153	8	CATCGCAC -> ATCGCACT	221 -> 226	Substitution	24.9% -> 27.6%

Contig 16

Name	Minimum	Maximum	Length	Change	Coverage	Polymorphism Type	Variant Frequency	Variant P-Value (approximate)	Amino Acid Change	CDS	product	Protein Effect	protein_id
	50291	50296	6	(CAGAGC)14 -> (CAGAGC)13	361 -> 362	Deletion (tandem repeat)	98.9% -> 99.2%	0	AEP -> A	chemotaxis protein CheW CDS	chemotaxis protein CheW	Deletion	KLI71480.1

GCC AGA	5037 9	5037 8	0	(CAGAGC)1 4 -> (CAGAGC)1 5	361 -> 368	Insertion (tandem repeat)	92.8% -> 92.9%	0	E -> EPE	chemotaxis protein CheW CDS	chemotaxis protein CheW	Inse rtion	KLI7 1480. 1

Contig 17

Name	Minimum	Maximum	Length	Change	Coverage	Polymorphism Type	Variant Frequency
G	22	22	1	A -> G	165	SNP (transition)	41.20%
A	36	36	1	G -> A	164	SNP (transition)	35.40%
C	72	73	2	AT -> C	163 -> 166	Deletion	30.7% -> 31.3%
A	216849	216848	0		98	Insertion	83.70%
T	216850	216850	1	A -> T	92	SNP (transversion)	89.10%
	216856	216857	2		357 -> 366	Deletion	53.3% -> 54.6%
A	216861	216861	1	T -> A	357	SNP (transversion)	38.70%
C	216862	216862	1	T -> C	354	SNP (transition)	54.00%
T	216864	216864	1	G -> T	354	SNP (transversion)	60.50%
T	216866	216866	1	G -> T	359	SNP (transversion)	53.50%
T	216868	216868	1	C -> T	364	SNP (transition)	53.00%
GA	216871	216872	2	TT -> GA	345 -> 348	Substitution	52.8% -> 53.2%
AA	216875	216875	1	C -> AA	329 -> 337	Insertion	46.6% -> 46.8%
T	216878	216878	1	C -> T	329	SNP (transition)	47.10%
G	216882	216882	1	T -> G	307	SNP (transversion)	49.80%

C	216946	216946	1	A -> C	213	SNP (transversion)	34.30%
	216955	216955	1		196	Deletion	26.50%
A	216958	216958	1	T -> A	189	SNP (transversion)	27.50%
G	217109	217109	1	A -> G	172	SNP (transition)	78.50%

Contig 18

Name	Minimum	Maximum	Length	Change	Coverage	Polymorphism Type	Variant Frequency
A	306	306	1	C -> A	183	SNP (transversion)	25.70%
A	308	308	1	G -> A	184	SNP (transition)	25.00%
GTAG	311	314	4	TCTT -> GTAG	187	Substitution	25.70%

Contig 19

Name	Minimum	Maximum	Length	Change	Coverage	Polymorphism Type	Variant Frequency
GC	96	97	2	CA -> GC	162 -> 163	Substitution	25.8% -> 25.9%
A	99	99	1	T -> A	168	SNP (transversion)	29.80%
T	197	197	1	C -> T	143	SNP (transition)	28.70%
CATTGTTATATCTCA	200	214	15	GCGGAGGCACATATT -> CATTGTTATATCTCA	112 -> 134	Substitution	25.9% -> 30.2%

AAGAGA	216	221	6	CGTTTT -> AAGAGA	103 -> 112	Substitution	25.2% -> 26.9%

Contig 23

Name	Minimum	Maximum	Length	Change	Coverage	Polymorphism Type	Variant Frequency
CT	2	3	2	TA -> CT	270 -> 271	Substitution	41.7% -> 41.9%
T	6	6	1	G -> T	278	SNP (transversion)	57.20%
T	68	68	1	G -> T	145	SNP (transversion)	53.80%
C	70	70	1	T -> C	145	SNP (transition)	53.80%
GTT	72	74	3	TGC -> GTT	145	Substitution	53.80%
C	78	78	1	A -> C	147	SNP (transversion)	62.60%
T	79	79	1	A -> T	147	SNP (transversion)	53.10%
T	80	80	1	G -> T	147	SNP (transversion)	62.60%
GC	82	83	2	TT -> GC	147 -> 148	Substitution	52.7% -> 53.1%
CAAAA	85	89	5	TCTCT -> CAAAA	147 -> 148	Substitution	52.0% -> 52.4%
GGA	92	94	3	AAG -> GGA	150	Substitution	51.30%
C	98	102	5	AGGTG -> C	150 -> 155	Deletion	50.3% -> 52.0%
T	107	107	1	C -> T	156	SNP (transition)	58.30%
G	114	114	1	T -> G	160	SNP (transversion)	48.80%
A	118	118	1	G -> A	160	SNP (transition)	48.10%

A	119	119	1	T -> A	160	SNP (transversion)	56.90%
G	120	120	1	T -> G	160	SNP (transversion)	47.50%
T	121	121	1	G -> T	160	SNP (transversion)	56.90%
T	132	132	1	A -> T	150	SNP (transversion)	44.70%
C	142	141	0	+C	131	Insertion	36.60%
TTC	147	149	3	AAT -> TTC	123 -> 124	Substitution	39.8% -> 40.3%
CAA	152	154	3	TTT -> CAA	114 -> 121	Substitution	33.3% -> 36.4%
CGTTTC	156	161	6	TAGAAG -> CGTTTC	93 -> 106	Substitution	29.1% -> 31.2%
GC	163	164	2	TT -> GC	90 -> 92	Substitution	26.7% -> 28.3%
G	167	167	1	T -> G	86	SNP (transversion)	26.70%

Contig 27

Name	Minimum	Maximum	Length	Change	Coverage	Polymorphism Type	Variant Frequency	CDS	product	Protein Effect	protein_id	Amino Acid Change
GCAACAGCAACAGCAACAGCAACAGCAACAGCAACA	27340	27339	0	(AACAGCAACAGCAACAGC)4 -> (AACAGCAACAGCAACAGC)6	114 -> 188	Insertion (tandem repeat)	86.4% -> 88.8%	hypothetical protein CDS	hypothetical protein	Insertion	KLI70436.1	Q -> QQQQQQQQQQQQQ

Contig 28

Name	Minimum	Maximum	Length	Change	Coverage	Polymorphism Type	Variant Frequency	Variant P-Value (approximate)	CDS	product	Protein Effect	protein_id
G	10120	10120	1	T -> G	1011	SNP (transversion)	47.50 %	0	calcium-binding protein CDS	calcium-binding protein	None	KLI70416.1
C	10129	10129	1	G -> C	987	SNP (transversion)	72.70 %	0	calcium-binding protein CDS	calcium-binding protein	None	KLI70416.1
T	10145	10145	1	C -> T	955	SNP (transition)	55.70 %	0	calcium-binding protein CDS	calcium-binding protein	None	KLI70416.1
C	10153	10153	1	T -> C	962	SNP (transition)	58.60 %	0	calcium-binding protein CDS	calcium-binding protein	None	KLI70416.1
T	10159	10159	1	C -> T	991	SNP (transition)	61.00 %	0	calcium-binding protein CDS	calcium-binding protein	None	KLI70416.1
A	10204	10204	1	C -> A	879	SNP (transversion)	33.90 %	0	calcium-binding protein CDS	calcium-binding protein	None	KLI70416.1
C	10273	10273	1	T -> C	992	SNP (transition)	43.00 %	0	calcium-binding protein CDS	calcium-binding protein	None	KLI70416.1

G	10282	10282	1	C -> G	1005	SNP (transversion)	43.20 %	0	calcium-binding protein CDS	calcium-binding protein	None	KLI70416.1
T	10322	10322	1	G -> T	1026	SNP (transversion)	27.10 %	0	calcium-binding protein CDS	calcium-binding protein	Substitution	KLI70416.1
T	10351	10351	1	A -> T	998	SNP (transversion)	35.70 %	0	calcium-binding protein CDS	calcium-binding protein	None	KLI70416.1
T	10360	10360	1	C -> T	1003	SNP (transition)	28.60 %	0	calcium-binding protein CDS	calcium-binding protein	None	KLI70416.1
A	10369	10369	1	G -> A	976	SNP (transition)	41.70 %	0	calcium-binding protein CDS	calcium-binding protein	None	KLI70416.1
A	10387	10387	1	G -> A	1003	SNP (transition)	41.30 %	0	calcium-binding protein CDS	calcium-binding protein	None	KLI70416.1
C	10396	10396	1	T -> C	1084	SNP (transition)	45.20 %	0	calcium-binding protein CDS	calcium-binding protein	None	KLI70416.1
G	10405	10405	1	C -> G	1056	SNP (transversion)	56.30 %	0	calcium-binding protein CDS	calcium-binding protein	None	KLI70416.1
G	10414	10414	1	A -> G	1050	SNP (transition)	34.50 %	0	calcium-binding protein CDS	calcium-binding protein	None	KLI70416.1

T	10429	10429	1	C -> T	1067	SNP (transition)	39.60 %	0	calcium-binding protein CDS	calcium-binding protein	None	KLI70416.1
T	10432	10432	1	C -> T	1069	SNP (transition)	27.50 %	0	calcium-binding protein CDS	calcium-binding protein	None	KLI70416.1
GT	10434	10435	2	CG -> GT	1067 -> 1069	Substitution	32.7% -> 32.9%	0	calcium-binding protein CDS	calcium-binding protein	Substitution	KLI70416.1
G	10441	10441	1	A -> G	1072	SNP (transition)	71.70 %	0	calcium-binding protein CDS	calcium-binding protein	None	KLI70416.1
C	10444	10444	1	A -> C	1099	SNP (transversion)	47.10 %	0	calcium-binding protein CDS	calcium-binding protein	None	KLI70416.1
T	10444	10444	1	A -> T	1099	SNP (transversion)	26.10 %	0	calcium-binding protein CDS	calcium-binding protein	None	KLI70416.1
C	10447	10447	1	T -> C	1104	SNP (transition)	26.30 %	0	calcium-binding protein CDS	calcium-binding protein	None	KLI70416.1
ACC	10481	10483	2	TCA -> ACC	1012 -> 1022	Substitution	49.6% -> 50.1%	0	calcium-binding protein CDS	calcium-binding protein	Substitution	KLI70416.1
C	10486	10486	1	T -> C	997	SNP (transition)	43.50 %	0	calcium-binding protein CDS	calcium-binding protein	None	KLI70416.1

C	10498	10498	1	T -> C	967	SNP (transition)	72.00%	0	calcium-binding protein CDS	calcium-binding protein	None	KLI70416.1
A	10534	10534	1	G -> A	63	SNP (transition)	87.30%	3.90E-167	calcium-binding protein CDS	calcium-binding protein	None	KLI70416.1
C	10540	10540	1	T -> C	54	SNP (transition)	90.70%	1.00E-165	calcium-binding protein CDS	calcium-binding protein	None	KLI70416.1
T	10546	10546	1	C -> T	45	SNP (transition)	80.00%	8.80E-118	calcium-binding protein CDS	calcium-binding protein	None	KLI70416.1
AG	10549	10550	2	GT -> AG	33 -> 34	Substitution	36.4% -> 38.2%	2.90E-37	calcium-binding protein CDS	calcium-binding protein	Substitution	KLI70416.1
AGCGGAAACG	10549	10558	9	GTCCACTGTT -> AGCGGAAACG	21 -> 34	Substitution	29.4% -> 43.5%	8.00E-18	calcium-binding protein CDS	calcium-binding protein	Substitution	KLI70416.1
G	10564	10564	1	C -> G	23	SNP (transversion)	34.80%	1.20E-16	calcium-binding protein CDS	calcium-binding protein	None	KLI70416.1

Contig 29

Name	Minimum	Maximum	Length	Change	Coverage	Polymorphism Type	Variant Frequency
G	133	133	1	A -> G	227	SNP (transition)	36.60%

T	135	135	1	A -> T	233	SNP (transversion)	35.60%
T	137	136	0		221	Insertion	27.60%
T	141	141	1	C -> T	207	SNP (transition)	30.00%
T	142	142	1	C -> T	206	SNP (transition)	40.30%
C	143	143	1	T -> C	206	SNP (transition)	53.90%
C	145	145	1	T -> C	207	SNP (transition)	52.70%
CG	147	148	2	TC -> CG	208 -> 210	Substitution	29.5% -> 29.8%
A	151	151	1	G -> A	206	SNP (transition)	39.30%
C	154	154	1	T -> C	206	SNP (transition)	41.70%
C	165	165	1	A -> C	186	SNP (transversion)	37.10%
T	168	168	1	G -> T	170	SNP (transversion)	53.50%
A	250	250	1	T -> A	147	SNP (transversion)	51.70%
AG	252	253	2	TT -> AG	146	Substitution	51.40%
TCTC	255	258	4	ATAA -> TCTC	144 -> 150	Substitution	48.0% -> 50.0%
C	260	260	1	T -> C	148	SNP (transition)	47.30%
GT	262	263	2	AA -> GT	144 -> 145	Substitution	46.5% -> 46.9%
C	265	265	1	G -> C	140	SNP (transversion)	48.60%
	266	279	14		137 -> 161	Deletion	33.5% -> 39.4%
T	281	281	1	C -> T	157	SNP (transition)	46.50%
GT	286	287	2	AA -> GT	169 -> 172	Substitution	44.8% -> 45.6%
C	290	290	1	T -> C	179	SNP (transition)	43.60%
AC	291	292	2	GA -> AC	181	Substitution	51.40%
AT	294	295	2	GC -> AT	194 -> 195	Substitution	37.9% -> 38.1%

	298	299	2		212	Deletion	35.80%

Contig 31

Name	Minimum	Maximum	Length	Change	Coverage	Polymorphism Type	Variant Frequency	Variant P-Value (approximate)	Amino Acid Change	CDS	product	Protein Effect	protein_id
	16050	16055	6	(TCTGCT)11 -> (TCTGCT)10	775	Deletion (tandem repeat)	99.00%	0	EAE -> E	cell division protein FtsY CDS	cell division protein FtsY	Deletion	KLI70360.1
TCTGCT	16116	16115	0	(TCTGCT)11 -> (TCTGCT)12	812 -> 826	Insertion (tandem repeat)	95.00%	0	P -> PAE	cell division protein FtsY CDS	cell division protein FtsY	Insertion	KLI70360.1

Contig 34

Name	Minimum	Maximum	Length	Change	Coverage	Polymorphism Type	Variant Frequency	Variant P-Value (approximate)	Amino Acid Change	CDS	product	Protein Effect	protein_id
G	3957	3957	1	A -> G	186	SNP (transition)	52.70%	6.80E-299		nucleoside permease CDS	nucleoside permease	None	KLI70272.1

AT	3996	3997	2	GC -> AT	156 -> 157	Substitution	50.0% -> 50.3%	1.10E-234	RH -> RY	nucleoside permease CDS	nucleoside permease	Substitution	KLI70272.1
G	4024	4024	1	T -> G	138	SNP (transversion)	43.50%	7.20E-177	E -> D	nucleoside permease CDS	nucleoside permease	Substitution	KLI70272.1
A	7429	7429	1	G -> A	162	SNP (transition)	25.30%	1.10E-109					
A	7436	7436	1	T -> A	161	SNP (transversion)	26.10%	1.50E-104					
AA	7441	7442	2	CG -> AA	158	Substitution	27.20%	3.20E-112					
A	7444	7444	1	C -> A	160	SNP (transversion)	26.90%	6.00E-112					
G	7448	7448	1	T -> G	163	SNP (transversion)	26.40%	7.60E-116					
A	7454	7454	1	C -> A	164	SNP (transversion)	26.80%	1.80E-114					
T	7474	7474	1	G -> T	159	SNP (transversion)	30.20%	1.30E-127					
A	7486	7486	1	G -> A	163	SNP (transition)	31.90%	3.40E-129					

Contig 38

Name	Minimum	Maximum	Length	Change	Coverage	Polymorphism Type	Variant Frequency
G	22	22	1	A -> G	212	SNP (transition)	31.10%

G	30	31	2	AA -> G	195 -> 198	Deletion	26.7% -> 27.3%
TGCT	35	38	4	AATC -> TGCT	186 -> 193	Substitution	25.3% -> 27.1%
C	41	41	1	G -> C	184	SNP (transversion)	25.50%

Contig 40

Name	Minimum	Maximum	Length	Change	Coverage	Polymorphism Type	Variant Frequency
C	66081	66081	1	G -> C	178	SNP (transversion)	43.30%
G	66087	66087	1	A -> G	176	SNP (transition)	43.80%
A	66092	66092	1	G -> A	175	SNP (transition)	44.00%
T	66114	66114	1	C -> T	169	SNP (transition)	45.60%

Contig 46

Name	Minimum	Maximum	Length	Change	Coverage	Polymorphism Type	Variant Frequency
A	1999	1999	1	C -> A	248	SNP (transversion)	31.50%
G	2012	2012	1	A -> G	247	SNP (transition)	27.50%
GCCC	2599	2602	4	ATTT -> GCCC	359 -> 365	Substitution	25.6% -> 26.0%
T	2604	2604	1	C -> T	360	SNP (transition)	26.10%
AGT	2607	2609	3	CTG -> AGT	362 -> 384	Substitution	26.2% -> 27.8%
A	2616	2616	1	G -> A	380	SNP (transition)	27.40%
T	2619	2619	1	A -> T	365	SNP (transversion)	28.50%

Contig 49

Name	Minimum	Maximum	Length	Change	Coverage	Polymorphism Type	Variant Frequency	Variant P-Value (approximate)	Amino Acid Change	CDS	product	Protein Effect	protein_id
C	8	8	1	G -> C	1375	SNP (transversion)	25.20%	0	D -> E	hypothetical protein CDS	hypothetical protein	Substitution	KLI70043.1
A	53	53	1	G -> A	894	SNP (transition)	32.90%	0		hypothetical protein CDS	hypothetical protein	None	KLI70043.1
G	59	59	1	A -> G	853	SNP (transition)	29.30%	0		hypothetical protein CDS	hypothetical protein	None	KLI70043.1
G	62	62	1	A -> G	828	SNP (transition)	27.50%	0		hypothetical protein CDS	hypothetical protein	None	KLI70043.1
GA	73	74	2	AG -> GA	801 -> 803	Substitution	25.6% -> 25.8%	0		hypothetical protein CDS	hypothetical protein	None	KLI70043.1
G	101	101	1	A -> G	971	SNP (transition)	41.50%	0		hypothetical protein CDS	hypothetical protein	None	KLI70043.1
CA	109	110	2	TC -> CA	938 -> 945	Substitution	38.0% -> 38.1%	0	AI -> AV	hypothetical protein CDS	hypothetical protein	Substitution	KLI70043.1
G	113	113	1	C -> G	913	SNP (transversion)	37.60%	0		hypothetical protein CDS	hypothetical protein	None	KLI70043.1
A	116	116	1	G -> A	886	SNP (transition)	37.10%	0		hypothetical protein CDS	hypothetical protein	None	KLI70043.1

CTT	121	123	3	TGG -> CTT	824 -> 854	Substitution	34.6% -> 35.7%	0	TN -> KD	hypothetical protein CDS	hypothetical protein	Substitution	KLI70043.1
T	125	125	1	C -> T	821	SNP (transition)	34.70%	0		hypothetical protein CDS	hypothetical protein	None	KLI70043.1
G	143	143	1	C -> G	810	SNP (transversion)	31.40%	0		hypothetical protein CDS	hypothetical protein	None	KLI70043.1
T	348	348	1	C -> T	1711	SNP (transition)	69.10%	0	G -> D	hypothetical protein CDS	hypothetical protein	Substitution	KLI70043.1
A	374	374	1	G -> A	1683	SNP (transition)	69.20%	0		hypothetical protein CDS	hypothetical protein	None	KLI70043.1
C	386	386	1	T -> C	1652	SNP (transition)	70.30%	0		hypothetical protein CDS	hypothetical protein	None	KLI70043.1
T	392	392	1	G -> T	2218	SNP (transversion)	77.50%	0	N -> K	hypothetical protein CDS	hypothetical protein	Substitution	KLI70043.1
A	395	395	1	G -> A	2213	SNP (transition)	65.70%	0		hypothetical protein CDS	hypothetical protein	None	KLI70043.1
A	407	407	1	G -> A	2124	SNP (transition)	33.40%	0		hypothetical protein CDS	hypothetical protein	None	KLI70043.1
C	413	413	1	T -> C	2099	SNP (transition)	75.10%	0		hypothetical protein CDS	hypothetical protein	None	KLI70043.1
T	416	416	1	A -> T	2039	SNP (transversion)	64.00%	0		hypothetical protein CDS	hypothetical protein	None	KLI70043.1
CGCT	419	422	3	GGTA -> CGCT	1939 -> 4249	Substitution	17.7% -> 38.8%	0	VT -> VA	hypothetical protein CDS	hypothetical protein	Substitution	KLI70043.1

CT	421	422	2	TA -> CT	1939 -> 4249	Substitution	27.1% -> 42.6%	0	VT -> VA	hypothetical protein CDS	hypothetical protein	Substitution	KLI70043.1
T	425	425	1	C -> T	4308	SNP (transition)	63.00%	0		hypothetical protein CDS	hypothetical protein	None	KLI70043.1
G	428	428	1	A -> G	4275	SNP (transition)	82.30%	0		hypothetical protein CDS	hypothetical protein	None	KLI70043.1
C	452	452	1	T -> C	3641	SNP (transition)	49.10%	0		hypothetical protein CDS	hypothetical protein	None	KLI70043.1
A	455	455	1	G -> A	3646	SNP (transition)	37.20%	0		hypothetical protein CDS	hypothetical protein	None	KLI70043.1
A	470	470	1	G -> A	3517	SNP (transition)	34.10%	0		hypothetical protein CDS	hypothetical protein	None	KLI70043.1
G	473	473	1	C -> G	3509	SNP (transversion)	51.80%	0		hypothetical protein CDS	hypothetical protein	None	KLI70043.1
G	479	479	1	C -> G	3482	SNP (transversion)	54.80%	0		hypothetical protein CDS	hypothetical protein	None	KLI70043.1
TAG	482	484	2	CAA -> TAG	3410 -> 3426	Substitution	25.9% -> 26.0%	0		hypothetical protein CDS	hypothetical protein	None	KLI70043.1
G	484	484	1	A -> G	3410	SNP (transition)	31.30%	0		hypothetical protein CDS	hypothetical protein	None	KLI70043.1
GGT	485	487	2	CGC -> GGT	3408 -> 3414	Substitution	25.50%	0	A -> T	hypothetical protein CDS	hypothetical protein	Substitution	KLI70043.1
C	509	509	1	G -> C	2780	SNP (transversion)	36.50%	0		hypothetical protein CDS	hypothetical protein	None	KLI70043.1

G	530	530	1	C -> G	4717	SNP (transversion)	35.40%	0		hypothetical protein CDS	hypothetical protein	None	KLI70043.1
A	563	563	1	G -> A	5010	SNP (transition)	31.70%	0		hypothetical protein CDS	hypothetical protein	None	KLI70043.1
A	608	608	1	G -> A	5144	SNP (transition)	41.40%	0		hypothetical protein CDS	hypothetical protein	None	KLI70043.1
G	614	614	1	A -> G	5105	SNP (transition)	33.20%	0		hypothetical protein CDS	hypothetical protein	None	KLI70043.1
T	623	623	1	C -> T	5180	SNP (transition)	26.60%	0		hypothetical protein CDS	hypothetical protein	None	KLI70043.1
C	628	628	1	T -> C	5060	SNP (transition)	42.00%	0	N -> D	hypothetical protein CDS	hypothetical protein	Substitution	KLI70043.1
G	671	671	1	A -> G	5212	SNP (transition)	33.00%	0		hypothetical protein CDS	hypothetical protein	None	KLI70043.1
T	675	675	1	C -> T	5085	SNP (transition)	25.30%	0	S -> N	hypothetical protein CDS	hypothetical protein	Substitution	KLI70043.1
A	677	677	1	G -> A	5114	SNP (transition)	39.20%	0		hypothetical protein CDS	hypothetical protein	None	KLI70043.1
A	722	722	1	G -> A	5483	SNP (transition)	26.30%	0		hypothetical protein CDS	hypothetical protein	None	KLI70043.1
T	728	728	1	C -> T	5444	SNP (transition)	25.50%	0		hypothetical protein CDS	hypothetical protein	None	KLI70043.1
A	737	737	1	G -> A	5487	SNP (transition)	72.80%	0		hypothetical protein CDS	hypothetical protein	None	KLI70043.1

C	752	752	1	G -> C	1840	SNP (transversion)	44.70%	0		hypothetical protein CDS	hypothetical protein	None	KLI70043.1
A	755	755	1	C -> A	1865	SNP (transversion)	45.10%	0		hypothetical protein CDS	hypothetical protein	None	KLI70043.1
G	806	806	1	C -> G	1894	SNP (transversion)	69.60%	0		hypothetical protein CDS	hypothetical protein	None	KLI70043.1
C	823	823	1	T -> C	1930	SNP (transition)	56.90%	0	I -> V	hypothetical protein CDS	hypothetical protein	Substitution	KLI70043.1
T	834	834	1	G -> T	1944	SNP (transversion)	67.50%	0	T -> K	hypothetical protein CDS	hypothetical protein	Substitution	KLI70043.1
C	844	844	1	T -> C	1412	SNP (transition)	66.80%	0	T -> A	hypothetical protein CDS	hypothetical protein	Substitution	KLI70043.1
A	854	854	1	G -> A	1387	SNP (transition)	66.30%	0		hypothetical protein CDS	hypothetical protein	None	KLI70043.1
A	881	881	1	G -> A	1452	SNP (transition)	60.50%	0		hypothetical protein CDS	hypothetical protein	None	KLI70043.1
A	887	887	1	G -> A	1456	SNP (transition)	65.90%	0		hypothetical protein CDS	hypothetical protein	None	KLI70043.1
G	896	896	1	A -> G	1486	SNP (transition)	67.00%	0		hypothetical protein CDS	hypothetical protein	None	KLI70043.1
CA	943	944	2	TC -> CA	774 -> 775	Substitution	36.7% -> 36.8%	0	AI -> AV	hypothetical protein CDS	hypothetical protein	Substitution	KLI70043.1
G	947	947	1	C -> G	794	SNP (transversion)	38.40%	0		hypothetical protein CDS	hypothetical protein	None	KLI70043.1

G	974	974	1	A -> G	1902	SNP (transition)	39.40%	0		hypothetical protein CDS	hypothetical protein	None	KLI70043.1
C	977	977	1	G -> C	1906	SNP (transversion)	41.30%	0		hypothetical protein CDS	hypothetical protein	None	KLI70043.1
C	983	983	1	T -> C	1956	SNP (transition)	62.40%	0		hypothetical protein CDS	hypothetical protein	None	KLI70043.1
C	986	986	1	G -> C	1964	SNP (transversion)	62.40%	0		hypothetical protein CDS	hypothetical protein	None	KLI70043.1
T	1013	1013	1	C -> T	2498	SNP (transition)	49.10%	0		hypothetical protein CDS	hypothetical protein	None	KLI70043.1
C	1016	1016	1	T -> C	2538	SNP (transition)	32.00%	0		hypothetical protein CDS	hypothetical protein	None	KLI70043.1
G	1025	1025	1	C -> G	2636	SNP (transversion)	26.40%	0		hypothetical protein CDS	hypothetical protein	None	KLI70043.1
T	1025	1025	1	C -> T	2636	SNP (transition)	49.00%	0		hypothetical protein CDS	hypothetical protein	None	KLI70043.1
T	1054	1054	1	C -> T	2945	SNP (transition)	60.10%	0	A -> T	hypothetical protein CDS	hypothetical protein	Substitution	KLI70043.1
A	1055	1055	1	C -> A	2950	SNP (transversion)	34.90%	0		hypothetical protein CDS	hypothetical protein	None	KLI70043.1
G	1055	1055	1	C -> G	2950	SNP (transversion)	41.70%	0		hypothetical protein CDS	hypothetical protein	None	KLI70043.1
A	1062	1062	1	T -> A	2947	SNP (transversion)	51.80%	0	Y -> F	hypothetical protein CDS	hypothetical protein	Substitution	KLI70043.1

Contig 53

Name	Minimum	Maximum	Length	Change	Coverage	Polymorphism Type	Variant Frequency
A	36	36	1	G -> A	347	SNP (transition)	31.70%
T	856	856	1	A -> T	6324	SNP (transversion)	26.60%
C	1196	1196	1	A -> C	5751	SNP (transversion)	50.50%
A	2748	2748	1	C -> A	5867	SNP (transversion)	32.90%
A	2757	2757	1	G -> A	5907	SNP (transition)	32.70%
C	2759	2759	1	T -> C	5796	SNP (transition)	32.90%

Contig 54

Name	Minimum	Maximum	Length	Change	Coverage	Polymorphism Type	Variant Frequency
G	61	61	1	A -> G	1275	SNP (transition)	32.20%
T	72	72	1	C -> T	1186	SNP (transition)	33.30%
G	1153	1153	1	A -> G	8706	SNP (transition)	34.40%
T	1172	1172	1	C -> T	8585	SNP (transition)	28.00%
C	1173	1173	1	T -> C	8488	SNP (transition)	43.40%
G	1550	1550	1	A -> G	4407	SNP (transition)	25.80%
A	1679	1678	0	(A)2 -> (A)3	1338	Insertion (tandem repeat)	39.00%
CGAA	1707	1710	4	TTTC -> CGAA	836 -> 846	Substitution	33.9% -> 34.1%

Contig 57

Name	Minimum	Maximum	Length	Change	Coverage	Polymorphism Type	Variant Frequency	Variant P-Value (approximate)	Amino Acid Change	CDS	product	Protein Effect	protein_id
G	159	159	1	T -> G	371	SNP (transversion)	59.60%	0		RTX toxin CDS	RTX toxin	None	KLI69968.1
G	173	173	1	A -> G	394	SNP (transition)	59.60%	0	D -> G	RTX toxin CDS	RTX toxin	Substitution	KLI69968.1
A	201	201	1	G -> A	421	SNP (transition)	40.10%	0		RTX toxin CDS	RTX toxin	None	KLI69968.1
G	363	363	1	A -> G	560	SNP (transition)	55.50%	0		RTX toxin CDS	RTX toxin	None	KLI69968.1
G	427	427	1	A -> G	545	SNP (transition)	52.80%	0	I -> V	RTX toxin CDS	RTX toxin	Substitution	KLI69968.1
C	437	437	1	T -> C	537	SNP (transition)	52.10%	0	L -> S	RTX toxin CDS	RTX toxin	Substitution	KLI69968.1
G	723	723	1	A -> G	648	SNP (transition)	57.60%	0		RTX toxin CDS	RTX toxin	None	KLI69968.1
G	728	728	1	T -> G	654	SNP (transversion)	56.40%	0	V -> G	RTX toxin CDS	RTX toxin	Substitution	KLI69968.1
A	745	745	1	G -> A	659	SNP (transition)	56.90%	0	A -> T	RTX toxin CDS	RTX toxin	Substitution	KLI69968.1

A	840	840	1	G -> A	618	SNP (transition)	56.80%	0		RTX toxin CDS	RTX toxin	None	KLI69968.1
C	986	986	1	T -> C	585	SNP (transition)	48.00%	0	I -> T	RTX toxin CDS	RTX toxin	Substitution	KLI69968.1
T	1019	1019	1	C -> T	594	SNP (transition)	49.30%	0	T -> M	RTX toxin CDS	RTX toxin	Substitution	KLI69968.1
C	1122	1122	1	A -> C	677	SNP (transversion)	47.30%	0		RTX toxin CDS	RTX toxin	None	KLI69968.1
A	1159	1159	1	G -> A	678	SNP (transition)	50.40%	0	A -> T	RTX toxin CDS	RTX toxin	Substitution	KLI69968.1
G	1191	1191	1	A -> G	646	SNP (transition)	50.20%	0		RTX toxin CDS	RTX toxin	None	KLI69968.1
T	1323	1323	1	C -> T	488	SNP (transition)	54.10%	0		RTX toxin CDS	RTX toxin	None	KLI69968.1
C	1353	1353	1	T -> C	459	SNP (transition)	55.80%	0		RTX toxin CDS	RTX toxin	None	KLI69968.1
T	1418	1418	1	C -> T	437	SNP (transition)	39.10%	0	S -> L	RTX toxin CDS	RTX toxin	Substitution	KLI69968.1
G	1425	1425	1	A -> G	427	SNP (transition)	37.90%	0		RTX toxin CDS	RTX toxin	None	KLI69968.1

Contig 59

Name	Minimum	Maximum	Length	Change	Coverage	Polymorphism Type	Variant Frequency
ACAAA	17	21	5	GTTGT -> ACAAA	106 -> 111	Substitution	50.0% -> 51.8%
T	22	22	1	G -> T	99	SNP (transversion)	69.70%
T	23	23	1	G -> T	99	SNP (transversion)	47.50%
TCA	25	27	3	CGG -> TCA	90 -> 96	Substitution	45.8% -> 46.7%
C	28	28	1	T -> C	88	SNP (transition)	64.80%
TA	29	30	2	AT -> TA	87 -> 88	Substitution	44.3% -> 44.8%
AC	33	34	2	TA -> AC	83	Substitution	45.80%
T	35	35	1	A -> T	83	SNP (transversion)	66.30%
T	36	36	1	G -> T	76	SNP (transversion)	50.00%
AG	37	38	2	GA -> AG	69 -> 74	Substitution	64.9% -> 68.1%
T	39	39	1	G -> T	67	SNP (transversion)	52.20%
T	41	41	1	A -> T	58	SNP (transversion)	65.50%
T	44	44	1	G -> T	57	SNP (transversion)	52.60%
A	45	45	1	T -> A	57	SNP (transversion)	64.90%
T	47	47	1	C -> T	44	SNP (transition)	38.60%
AT	48	49	2	GA -> AT	36 -> 38	Substitution	47.4% -> 50.0%

Contig 60

Name	Minimum	Maximum	Length	Change	Coverage	Polymorphism Type	Variant Frequency
C	98	98	1	T -> C	122	SNP (transition)	43.40%

Contig 62

Name	Minimum	Maximum	Length	Change	Coverage	Polymorphism Type	Variant Frequency
CG	2723	2724	2	TT -> CG	30	Substitution	96.70%
T	2728	2728	1	C -> T	29	SNP (transition)	100.00%
T	2744	2744	1	A -> T	23	SNP (transversion)	100.00%
A	2747	2747	1	G -> A	22	SNP (transition)	100.00%

Contig 63

Name	Minimum	Maximum	Length	Change	Coverage	Polymorphism Type	Variant Frequency	Variant P-Value (approximate)	Amino Acid Change	CDS	product	Protein Effect	protein_id
GCAACA GCAACA	89	88	0	(AGCAAC)14 -> (AGCAAC)16	193 -> 199	Insertion (tandem repeat)	100.00%	0	Q -> QQQQQ	hypothetical protein CDS	hypothetical protein	Insertion	KLI69899.1
